# Integrating the DNA damage and protein stress responses during cancer development and treatment

**DOI:** 10.1002/path.5097

**Published:** 2018-07-19

**Authors:** Vassilis G Gorgoulis, Dafni‐Eleftheria Pefani, Ioannis S Pateras, Ioannis P Trougakos

**Affiliations:** ^1^ Molecular Carcinogenesis Group, Department of Histology and Embryology, School of Medicine National and Kapodistrian University of Athens Athens Greece; ^2^ Biomedical Research Foundation of the Academy of Athens Athens Greece; ^3^ Faculty of Biology, Medicine and Health University of Manchester, Manchester Academic Health Science Centre Manchester UK; ^4^ CRUK/MRC Institute for Radiation Oncology, Department of Oncology University of Oxford Oxford UK; ^5^ Department of Cell Biology and Biophysics, Faculty of Biology National and Kapodistrian University of Athens Athens Greece

**Keywords:** homeostasis, stress response, cancer, DNA damage response, proteome damage response, oncogenes, tumor suppressors, replication stress

## Abstract

During evolution, cells have developed a wide spectrum of stress response modules to ensure homeostasis. The genome and proteome damage response pathways constitute the pillars of this interwoven ‘defensive’ network. Consequently, the deregulation of these pathways correlates with ageing and various pathophysiological states, including cancer. In the present review, we highlight: (1) the structure of the genome and proteome damage response pathways; (2) their functional crosstalk; and (3) the conditions under which they predispose to cancer. Within this context, we emphasize the role of oncogene‐induced DNA damage as a driving force that shapes the cellular landscape for the emergence of the various hallmarks of cancer. We also discuss potential means to exploit key cancer‐related alterations of the genome and proteome damage response pathways in order to develop novel efficient therapeutic modalities. © 2018 The Authors. *The Journal of Pathology* published by John Wiley & Sons Ltd on behalf of Pathological Society of Great Britain and Ireland.

## Introduction: homeostasis and stress response

**Figure 1:**
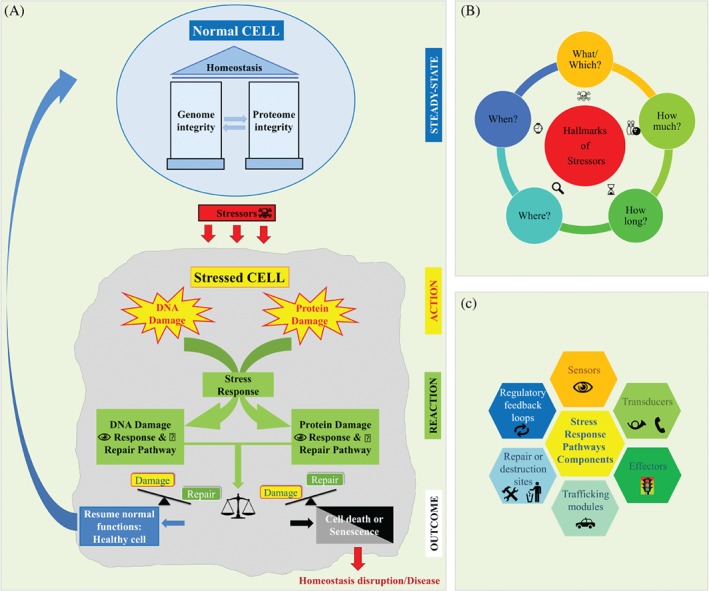
Overview of homeostatic mechanisms. (A) DDRs and PDRs. (B) Hallmarks of stressors. (C) SRP components. See supplementary material for a detailed legend.

The human body is continually exposed to a variety of stressors, a generic/collective term describing exogenous and/or endogenous noxious events/factors that disrupt the steady state of the cell 1. The cell's tendency to resist changes in order to maintain the status quo is called homeostasis [derived from the Greek words *homeo* (same) and *histimi* (standing up)] 2. From a thermodynamic point of view, living organisms are open systems that are constantly pushed away from their equilibrium. This measure of disorder or ataxia is termed entropy, and represents ‘the general trend of the universe towards death and disorder’, as stated by Newman 3. When a stressor ‘enters the scene’, this delicate balance between order and disorder tilts towards pathophysiological states, if not dealt with efficiently (Figure 1A). To preserve homeostasis and counteract such challenges, cells have developed the capacity to mount a multiplicity of stress responses (SRs) 4. Remarkably, reconstruction of the phylogeny of SRs makes it apparent that many of the key players participating in SRs are not only evolutionarily conserved, but still serve related functions.

Both the nature and the end result of a SR are determined by the type (what/which), magnitude (how much) and duration (how long) of the deleterious stimulus. Additionally, the spatiotemporal parameters of where (tissue organ/cellular/subcellular site) and when (young, old, or stage of a pathophysiogical process – early or late) the stressogenic event takes place constitute the parameters that affect the final outcome in a particular cellular context (Figure 1B). Although all biomolecules are susceptible to damage, dysfunction of the genome and protein machineries will mainly affect the cell's fate. Highly sophisticated and complex protein networks curate and maintain the integrity of the genome and proteome, comprising the so‐called DNA damage response (DDR) and protein damage response (PDR) pathways 5, 6. These damage/stress response pathways (SRPs) are hierarchically constructed and composed of sensors, signalling cascades (transducers and effectors), trafficking and repair modules, or destruction sites (Figure 1C). Each step/process is regulated by regulatory feedback loops that ensure an efficient and fine‐tuned response, which is gradually turned off once the damage is repaired, or proceeds to cell death if the damage is irreversible. However, things are not always so clear. Depending on the SR parameters (Figure 1B), there are variations in the way in which the cell orchestrates its reaction (supplementary material, Figure S1). For instance, a high level of damage in a low bioenergetic state (deprivation of ATP resources) will trigger necrosis instead of apoptosis, which is energy‐based 7. Necrosis predisposes to inflammation, whereas apoptosis is a ‘benign and inflammation‐free’ process 8. Then again, moderate DNA damage persisting because of repair difficulties, in an energy‐proficient environment, can trigger a prolonged cell cycle arrest called senescence* (see the Glossary in the supplementary material for further information on items marked with an asterisk). Senescence has a bright side and a dark side; it can operate as a tumor barrier, but can also foster a protumorigenic environment by resulting in the secretion of a broad spectrum of growth factors and cytokines (SASP*) 9, 10. As senescent cells are resistant to apoptosis, clearance by the immune system will neutralize long‐term harmful effects induced by SASP 11. Concurrently, as energy supplies are consumed, autophagy* is induced to secure cellular fitness, and restore metabolism and homeostasis 12, 13. In contrast, low levels (how much) of persistent (how long) DNA damage may have a long‐term beneficial effect, termed hormesis 14, recalling Nietzsche's quote ‘That which does not kill us, makes us stronger.’

Moreover, RNAs have an active role in SRs. They are considered to be the ancestral life‐encoding molecules 15, and are heterogeneous and versatile molecules that regulate many cellular processes 16. They include long and short RNAs, as well as coding and non‐coding RNAs, with the last group being in the majority 17. RNAs are critical in the genome reaction to SRs. They are involved in development and normal cell physiology, as well as in disease states, including cancer (supplementary material, Table S1), and other SRs 16. From the pathologist's point of view, the cellular reaction to SRs at the RNA level is illustrated by the generation of stress granules, which are dense cytosolic aggregations lacking a membrane, composed of RNAs and proteins 18. These granules store and protect RNAs in stressed cells, and are mainly identified by use of the TIA‐1* and G3BP1* factors 19. Detailed information on this field is provided in other excellent reviews (see supplementary material, Table S1).

Notably, in metazoans, such as humans, the response to stress is not limited to the cellular (local) level, but, importantly, is also elicited intercellularly/systematically (global). This intercellular response is orchestrated mainly via the immune system. It is a paracrine and circulation‐based response that attracts the diverse immune cell types at the sites of ‘damage’ in an attempt to ‘repair and clear it’. The immune cells execute specific and highly contextualized transcriptional programmes promoting immunity, while maintaining homeostasis. Remarkably, >1600 genes are involved in innate and adaptive immune responses 20, 21. The immune system is a constantly evolving functional entity via mechanisms of adaptation, expansion, maturation, and, ultimately, immunological memory, and represents the cornerstone of stewardship at the organismal level 22, 23, 24.

Consequently, the outcome of this ‘stressor versus SR confrontation’ will be determined by the cellular, microenvironmental and organismal context, leading either to healing or to various pathophysiological conditions. In most pathological situations, quantitative and/or qualitative defects in the DDR and PDR pathways are almost always observed (supplementary material, Figure S1). In the case of DDR, the significance of such alterations is underscored by the wide spectrum of clinical manifestations that occur when its components are defective (supplementary material, Table S2). Among the most unfavourable consequences of DDR and PDR deregulation is predisposition to cancer. Cancer is a complex, heterogeneous disease with an adverse prognosis if not diagnosed early. It evolves in a multistep process, and each phenotypic stage reflects a particular set of molecular alterations that provides a selective advantage to the tumor cells carrying them. Depending on cancer type, diverse molecular defects may occur, although key events, such as p53 mutations or pRb pathway inactivation, are common among malignancies 25, 26. Deciphering these molecular dynamics will help us to better understand the trails of carcinogenesis, and will aid in the development of efficient therapeutic strategies. Therefore, mutational signatures* identified by analysing the genomes of various tumors represent the ‘DNA repair traits’, and can help to unveil the repair routes engaged during carcinogenesis, thus suggesting potential therapeutic targets 27, 28. These ‘DNA repair traits’ form part of a broader process termed genomic instability.

## DDR–PDR: an integrated genome–proteome maintenance network

**Figure 2:**
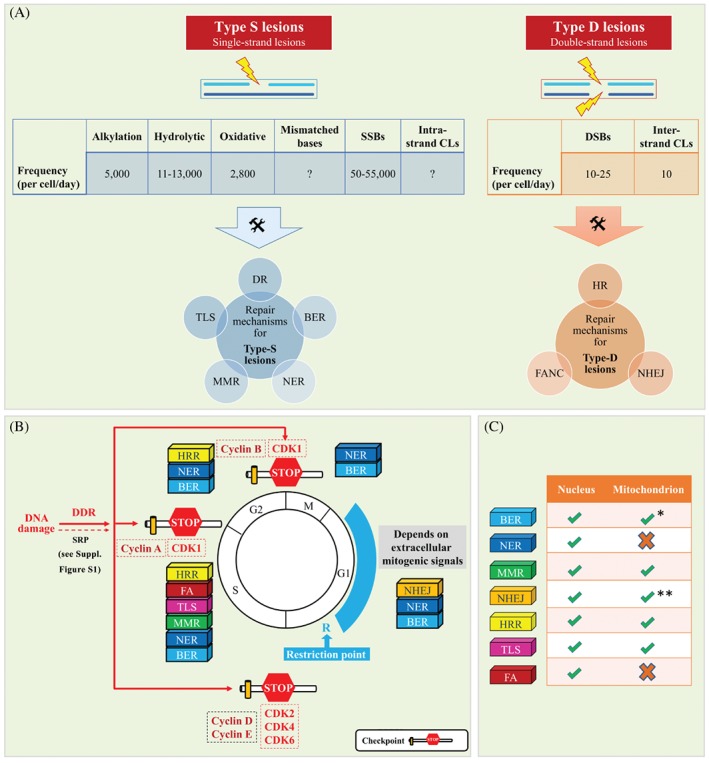
Synopsis of DNA damage type frequency and repair (A), DDR signalling cascades that activate the various checkpoints (B), and DNA repair mechanisms (C). See supplementary material for a detailed legend.

**Figure 3:**
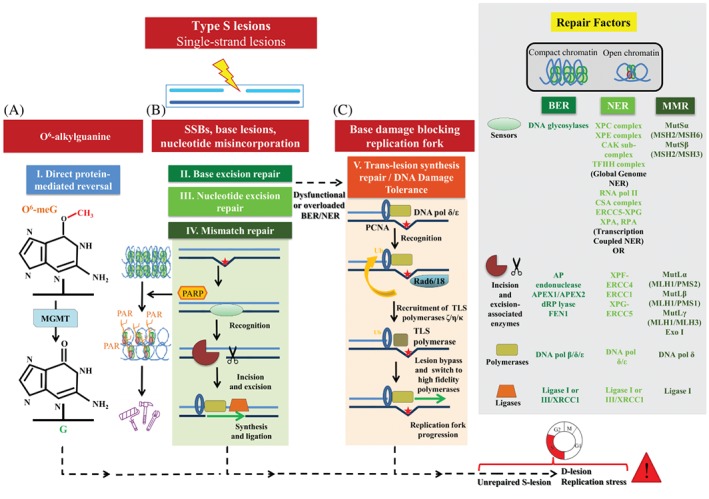
Repair routes for category‐S lesions (DDR surveillance). (A) Direct protein‐mediated reversal. (B) BER and NER. (C) TLS repair. See supplementary material for a detailed legend.

**Figure 4:**
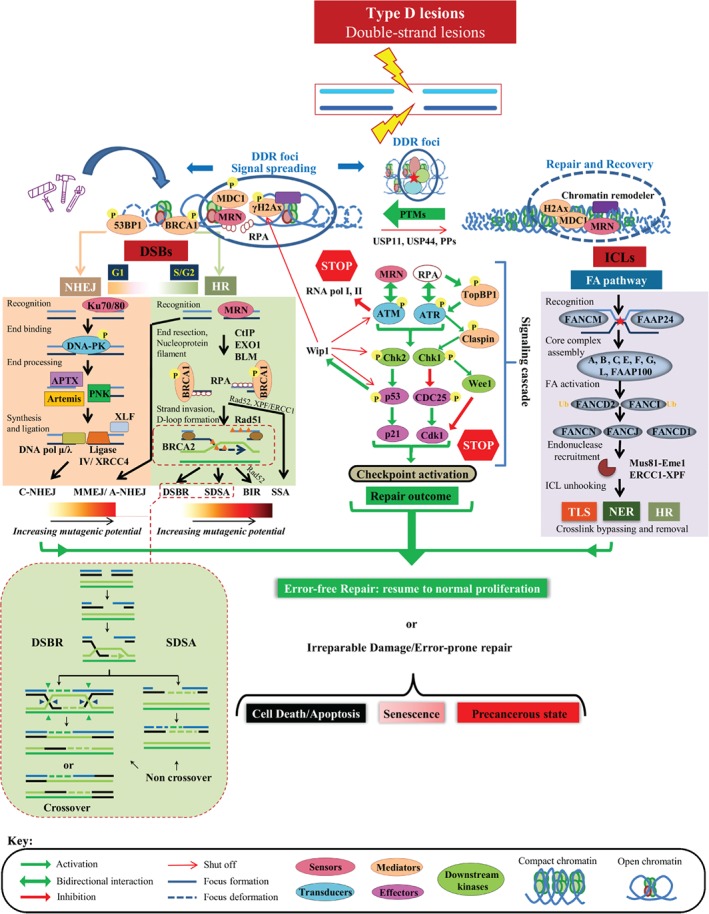
Repairing category‐D lesions (DDR surveillance). See supplementary material for a detailed legend.

GI* is a feature that nearly all tumors share, and is considered to be a hallmark of cancer 29. By the term GI, we refer to the high frequency of genetic alterations within the genome of a cellular lineage. Genetic alterations can range from single‐nucleotide substitutions (SNSs) (point mutations) to more complex quantitative and/or qualitative changes such as chromosome losses, gains, and/or rearrangements. GI can result from malfunctions at various steps of the DNA cycle, from replication to chromosome segregation 30, 31. The corollary of GI is that the transcriptome and proteome landscapes will progressively change, affecting the functionality of the cell. To comprehend the nature of this abnormal, albeit dynamic, process, we need to go through the essential operational characteristics of the DDR and PDR pathways and how they are physiologically wired and rewired during cancer development (Figures 2, 3, 4).

### Overview of the DNA damage response and repair (DDR/R) network

As DNA is the repository of genetic information, the ultimate goal of the DDR/R network is to preserve its integrity. However, because of the large number of DNA lesions induced in a cell every day, this task is not always achieved without cost (Figure 2) 32, 33. DNA lesions can be divided in two broad categories: those occurring on one strand of the double helix (category‐S lesions), such as modified bases, abasic sites, helix‐distorting base lesions, and single‐strand breaks; and those involving both strands [category‐D lesions: interstrand crosslinks and double‐strand breaks (DSBs)]. In certain cases, such as exposure to ionizing radiation, category‐S and/or category‐D lesions coexist, and, when they are in close vicinity, they are termed clustered DNA lesions* 34. As shown in Figure 2, the type of DNA lesion and the cell‐cycle phase largely dictate the repair programme to be engaged. Whereas some types of damage, such as *O* ^6^‐methylguanine, are subjected to direct protein‐mediated reversal (Figure 3A), most are repaired by a series of catalytic events entailing multiple proteins and generally including two steps: (1) damage recognition by sensors; and (2) processing and repair of the lesion (Figures 3B and 4). Depending on which damage category is to be repaired, these steps encompass unique characteristics.

### Repairing category‐S lesions

For category‐S lesions, subsequent to recognition, the following are required for processing and repair: (1) incisions flanking the damage; (2) excision of the damaged area; (3) filling of the gap by nucleotide polymerization; and (4) ‘sealing’ the gap with ligation (Figure 3). More specifically, the high‐fidelity (error‐free) pathways base excision repair (BER) 35, 36 and nucleotide excision repair (NER) 37 deal with single‐base DNA defects and helix‐disorting base lesions, respectively, whereas repair of nucleotide misincorporation is mediated by mismatch repair (MMR) (Figure 3B) 38. In the case of NER, the global release of RNAPII waves from promoter proximal pausing sites maximizes sensing and accelerates the repair of category‐S lesions equally well in genes with low and high expression via transcription‐coupled (TC) NER, guaranteeing unbiased transcriptome surveillance 39. However, if BER and NER malfunction or are overloaded by ‘fixing’ demands, then the translesion synthesis (TLS) pathway, which is a low‐fidelity repair module (error‐prone) pathway known as DNA‐damage tolerance (DDT), takes over (Figure 3C) 40. To avoid replication of damaged DNA that could lead to fork collapse, DSB production, and genome destabilization, cells opt to recruit TLS/DDT to bypass encountered lesions and repair them at a later time 40. Thus, TLS/DDT is considered to be responsible for the majority of mutagenic events, playing a central role in carcinogenesis. Although the latter is an undesired event, from a broader perspective it is a ‘cost’ that the cell has to pay to avoid DSBs, thus preserving double helix continuity 41, 42. In line with this, inhibition of factors involved in category‐S defect repair processes has the potential to induce DSB formation during replication, triggering RS* and death if the cell is also deficient in components implicated in DSB repair (Figure 3). One of the best examples supporting this scenario is the enzyme poly(ADP‐ribose) polymerase (PARP) 43. PARP is a vital repair protein involved in single‐strand break (SSB) repair and BER. PARP catalyses the synthesis of negatively charged poly(ADP‐ribose) chains by utilizing the respiratory coenzyme NAD^+^, with release of nicotinamide. The negative charge of the ADP‐ribose polymers around SSBs repels the positively charged histones of the nucleosomes, opening chromatin and thus allowing access to the repair machinery 8. Targeting of PARP has been shown to kill cancer cells deficient in the homologous recombination factors breast cancer susceptibility gene 1 (BRCA1) and breast cancer susceptibility gene 2 (BRCA2) (see below), paving the way for the use of PARP inhibitors in clinical practice 44, 45.

### Repairing category‐D lesions

DSBs are category‐D defects, and are considered to be the most deleterious types of DNA damage. From their frequency (Figure 2), it is apparent that the cell cannot tolerate them, as repair of a single DSB requires >10^5^ ATP molecules 46. Such an energy investment calls for a staggering level of cellular reorganization once DSBs occur. With the exception of immune receptor diversity [V(D)J and class switch (CS) recombination] and chromosomal crossover during meiosis II of gamete production, in which DSBs form part of physiological programmes 47, 48, the cellular reaction to DSBs epitomizes an integrated cellular SR to ‘imminent danger’. Once a DSB is formed, a cascade of biochemical events take place within minutes in an effort to efficiently ‘access, repair, and restore’ 49, 50. These biochemical processes are characterized by extensive PTMs* of the involved DDR proteins and chromatin structure of the damaged area; these processes: (1) are much faster chemical reactions than transcription; and (2) form docking sites for the recruitment of repair factors 51.

Two classes of DDR proteins are recruited at damaged sites: those that present directly at DSBs (called sensors* and mediators*), and those associated with the DSB‐flanking chromatin, altogether constituting so‐called DDR foci (Figure 4) 52. Over time, the DDR foci spread away from the DSB to distances up to megabases in mammals, forming an amplification mechanism recruiting signal transduction factors that further amplify the signal with effectors that set the cell in an ‘alarm’ state (Figure 4; DDR cascade). This mechanism has, on the one hand, a local effect by relaxing the chromatin and increasing the concentration of repair factors at the damaged site, and, on the other hand, a systemic effect, termed checkpoint activation*, that reduces the activity of CDKs* (Figure 2B) 5. Notably, in certain cases and depending on the cellular context, checkpoint activation, apart from the DDR signalling cascade, also involves the parallel action of other SR signalling routes (Figure 2), like the p38 mitogen‐activated protein kinase (p38MAPK) pathway, which coordinates several cellular functions 53. The endpoint of the SR signalling cascade is always the cyclin–CDK complex* (Figure 2B). The cyclin–CDK complexes represent drivers of the cell cycle and, when they are suppressed, the cell enters a state of arrest, providing time for repair.

One of the earliest features that mark these DDR foci is histone variant H2AX phosphorylation at serine 139, (also referred to as γH2AX), by ataxia telangiectasia mutated (ATM) backed up by ataxia telangiectasia and Rad3‐related (ATR) and DNA‐dependent protein kinase, catalytic subunit (DNA‐PK); all three kinases are members of the phosphatidylinositol 3‐kinase (PI3K) family and key DDR signalling components (transducers) 51, 54, 55, 56, 57. Subsequently, the DNA damage mediator called mediator of DNA‐damage checkpoint 1 (MDC1) attaches to γH2AX, acting as a platform for the meiotic recombination 11 (MRE11)–Rad50–Nijmegen breakage syndrome 1 (NBS1) (MRN) sensor complex that activates ATM, thus forming an amplification loop 58, 59. Concurrently, MRN complexes bind the DSB avidly, playing a pivotal role in the initial processing of the break, generating single‐strand (ssDNA) DNA 3′‐overhangs that are recognized by replication protein A (RPA). This event brings into play the ATR transducer kinase, which, in cooperation with ATM, turns on the downstream transducer* kinases checkpoint kinase 1 (Chk1) and checkpoint kinase 2 (Chk2) (Figure 4) 60. In concert, these kinases activate a key effector* of the DDR pathway, namely p53 61, 62. p53 is a transcription factor that governs a complex SR programme covering a bewildering range of biological functions, explaining why p53 is frequently mutated in cancer 63. Activation of p53, mainly via PTMs, induces the expression of numerous downstream effectors, including the universal CDK inhibitor p21^WAF1/Cip1^, leading to cell‐cycle arrest. Concurrently, ATM imposes a transcriptional silencing programme by shutting down both RNA polymerase II* and RNA polymerase I*, thus saving the energy that transcription demands and preventing collision between transcription and repair (analysed below in ‘Malfunction of endogenous operations as a cause of DNA damage’) 64, 65, 66. In parallel with this, the cell gains time and reshuffles energy resources for the repair machinery to ‘seal’ the DSBs, or it otherwise proceeds to apoptosis or senescence, depending on the extent of damage (Figure 4; supplementary material, Figure S1). Concomitantly with these global effects, repair is facilitated by extensive chromatin modifications and remodelling* at the site of the DSB 67. In brief, SWI/SNF‐dependent histone H2A.Z exchange for histone H2A destabilizes the nucleosomes surrounding the DSB. This nucleosome remodelling event exposes the N‐terminal tail of histone H4, which, in turn, is acetylated by TIP60 histone acetyltransferase, further relaxing chromatin and enabling access to downstream repair factors 68.

**Figure 5:**
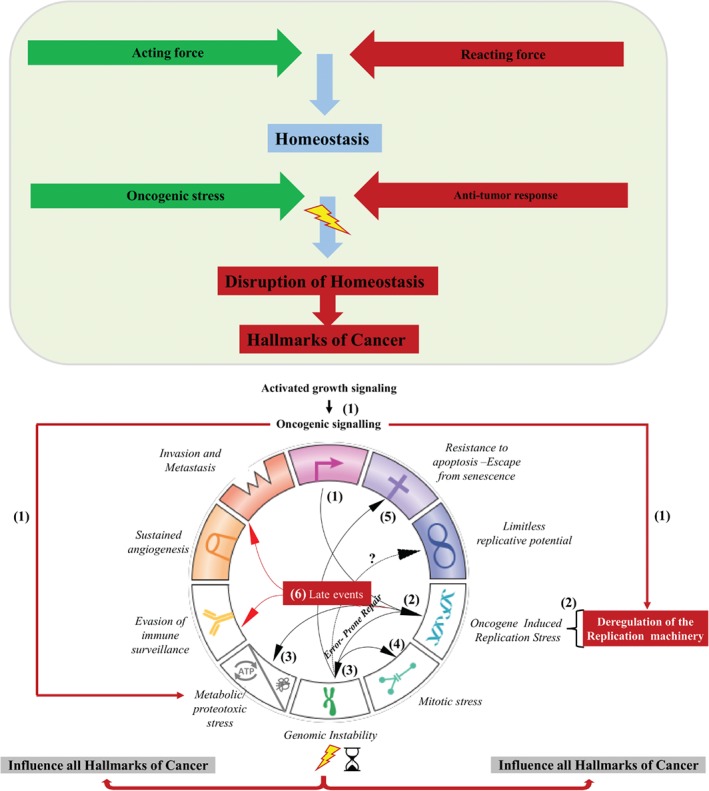
A model depicting how oncogene‐induced RS aids the progressive formation of certain hallmarks of cancer (early events: steps 1–5), while paving the way for angiogenesis, evasion from immune surveillance, invasion, and metastasis (late events: step 6). See supplementary material for a detailed legend.

The actual repair of the DSB lesion is carried out by homologous recombination repair (HRR) and non‐homologous end joining (NHEJ) (Figure 4; supplementary material, Figure S1) 69, 70. HRR is considered to be an error‐free repair system occurring during the S and G_2_ cell‐cycle phases, whereas NHEJ is an error‐prone repair pathway dealing mainly with non‐replication‐associated DSBs, and represents the predominant repair route that functions irrespective of the cell cycle (Figure 2). HRR is initiated by the binding of BRCA1 to the ubiquitin chain added by the E3 ligases ring finger protein 8 (RNF8) and ring finger protein 168 (RNF168) to the remodelled nucleosome 49, 71, 72, 73. In this way the BASC* connects sensing and signalling with the repair component BRCA2, which controls the Rad51 recombinase that replaces RPA. The BRCA2–Rad51 complex then invades the homologous template and primes DNA synthesis, copying and restoring the genetic information 74, 75. When the homologue donor strand is the sister chromatid, HRR is accurate. However, recombination may take place across different genome regions, challenging previous notions concerning the error‐free nature of HRR (Figure 5) 76. Hence, to secure sealing of DSBs, various routes of HRR exist that may favour inappropriate pairing. Alternatively, three critical histone modifications, namely histone H4 lysine 20 dimethylation (H4K20^me2^) [catalysed sequentially by methyltransferases SU(VAR)3‐9H1 and SETD8], ubiquitylation of histone H2A on lysine 15 (H2AK15^ub^) (induced by the E3 ligase RNF168), and histone H3 lysine 79 methylation (H3K79^me^), are recognized by the signalling mediator p53‐binding protein 1 (53BP1) at DSBs, promoting NHEJ 77, 78, 79, 80, 81, 82. Importantly, 53BP1 accumulation antagonizes BRCA1‐mediated HRR in favour of NHEJ (Figure 4) 83, 84, 85. In NHEJ, the DSB is sensed by the lupus Ku autoantigen protein p80 (Ku80)–lupus Ku autoantigen protein p70 (Ku70) heterodimer, which recruits and assembles the DNA–PK complex, which, in turn, processes the DNA ends and increases the recruitment of ligase IV/X‐ray repair complementing defective repair in Chinese hamster cells 4 (XRCC4), which carries out the rejoining reaction 74. Although HRR is the favoured pathway to deal with a DSB, ensuring DNA sequence fidelity, in the event that HRR is non‐functional the cell ‘prefers’ to follow inappropriate repair routes to secure cell viability 86. In this case, the faster kinetics of the Ku heterodimer for DSBs compared to those of the HRR factors 87 make the error‐prone NHEJ (the repair pathway of choice) operate even during S phase, with potential unfavourable effects for the cell 86, 88, 89, 90.

The second type of category‐D defects comprises DNA interstrand crosslinks (ICLs) that are generated by a class of agents such as mitomycin C (MMC) and diepoxybutane (DEB), or circulating metabolites such as formaldehyde. ICLs are toxic, because the covalent links that they form prevent DNA from unwinding, thereby blocking replication and transcription, causing replication and transcription stress, respectively. These lesions are fixed by the Fanconi anaemia (FA) pathway, which is a replication‐dependent repair mechanism that appeared relatively late in evolution (Figure 4) 91. It is considered to be the most sophisticated repair route, enlisting modules of three classic repair pathways, i.e. TLS, NER, and HRR. The central components of the FA pathway are the 13 complementation groups identified so far, i.e. Fanconi anaemia complementation group (FANC) A, FANCB, FANCC, and FANCD1 (better known as BRCA2), FANCD2, FANCE, FANCF, FANCG, FAQNCI, and FANCJ [BRCA1‐interacting protein C‐terminal helicase 1 (BRIP1) or BTB domain and CNC homologue 1 (BACH1)], and FANCL, FANCM, and FANCN [also termed partner and localizer of BRCA2 (PALB2)], which are mutated with various frequencies in FA* 92. FANCA–FANCG, FANCC–FANCE–FANCF and FANCB–FANCL–FAAP100 form the core complex that is recruited at the damage site by FANCM and FA‐associated protein of 24 kDa (FAAP24), which sense the stalled replication fork. The FA core complex* possesses ubiquitin ligase properties; it mono‐ubiquitinates FANCD2 and FANCI, which is regarded as the essential step for FA activation, in analogy to proliferating cell nuclear antigen (PCNA) ubiquitination‐dependent recruitment of TLS polymerases. Subsequently, DNA repair is organized by engaging ubiquitinated FANCD2 and FANCI with the downstream factors FANCD1 (BRCA2), the helicase FANCJ (BRIP1 or BACH1) and BRCA2 (FANCD1)‐interacting partner FANCN, also termed PALB2 93. The subsequent repair steps require coordinated and sequential activity of TLS, NER and HR enzymes. Once the replication fork moving from both directions stalls at the ICL, a dual incision by endonucleases methyl methanesulphonate and ultraviolet (UV)‐sensitive clone 81 (MUS81) and essential meiotic structure‐specific endonuclease 1 (EME1) at the 3′‐end of the lesion, and excision repair cross‐complementation group 1 (ERCC1) (XPF) at the 5′‐end unhooks the ICL, which is then bypassed by a TLS polymerase, probably Rev1 (polymerase ζ). NER removes the bypassed crosslink and HRR repairs the broken chromatid by using the newly repaired by TLS sister as a template (Figure 4) 37, 94.

### Shutting off the DDR pathway

In a normal setting, DDR/R activation is coupled with inactivation to allow a cell to complete its cycle. Feedback control of DDR fine‐tunes the magnitude and duration of the response, limiting aberrant DNA repair. Turning off DDR/R is a more arcane process than it was initially considered to be. Timely termination of DDR is controlled in a spatiotemporal manner, by different mechanisms acting in an overlapping manner 95. As activation of DDR/R signalling encompasses a series of PTMs, shutting it off calls for the reverse procedure. To date, several protein phosphatases (PPs) and deubiquitinating enzymes (DUBs) have been recognized as critical players in DDR/R termination (Figure 4). PP2A is among the best studied PPs; it catalyses dephosphorylation of γH2AX 96. PP2 catalytic subunit‐α (PP2A) inhibition results in persistent γH2AX foci, compromising DDR/R and rendering cells hypersensitive to DNA damage. Another DDR/R regulator is Wip1, which is involved in the dephosphorylation of multiple DDR components 97, 98. Notably, Wip1 is a transcriptional target of p53, and is stimulated after genotoxic stress 99. Activation of Wip1 100 facilitates p53 degradation by murine double minute 2 (MDM2), forming a negative feedback loop that allows termination of DDR. Conversely, overexpression of Wip1 has an oncogenic effect, signifying the importance of fine‐tuning for proper DDR/R operation (Figure 4) 98, 101. Recruitment of ubiquitin‐specific peptidase (USP) 44 and USP11 at DSBs antagonizing RNF8/RNF168 mono‐ubiquitination of histone H2A 102, 103 represents an example of how DUBs regulate DDR/R. Likewise, the activity of the deubiquitinating complex USP1–UAF1 over FANCD2 keeps the FA pathway in check 104, 105.

An additional way to terminate the activity of phosphorylated checkpoint proteins is through proteolytic degradation 106. Phosphorylation marks targets for proteolysis via the ubiquitin–proteasome (UPP) pathway, pinpointing the interdependence of the proteostasis and DDR/R networks 107. This mechanism is exemplified by SCF^βTrcP^, which acts as a switch between checkpoint initiation and recovery 6. Upon DNA damage, phosphorylated CDC25A is recognized by SCF^βTrcP^, promoting G_2_ arrest, whereas, when the DNA damage is repaired, the same complex mediates claspin* and Wee1* degradation, favouring checkpoint recovery and progression to mitosis.

### Malfunction of endogenous operations as a cause of DNA damage

Further to various exogenous mutagenic agents (clastogens*), deregulation of endogenous processes can create a genetic landscape prone to DSBs, adding an extra level of pathophysiological complexity. As with exogenous stimuli, a key aspect related to endogenous operations is keeping CDK activity in check. In mammals, several CDK–cyclin complexes are essential for normal proliferation 108. The significance of these complexes is underscored by the fact that aberrant CDK activity is a universal feature of human tumors. Deranged function of cyclin–CDK complexes 108 is counteracted by checkpoint activation, which is aimed at preserving genome integrity (Figure 2) 5, 109. Distinct checkpoints halt the cell cycle at specific phases and activate the DDR/R pathway, allowing time for DNA repair completion before entry into mitosis (Figure 4) 6. If there is extensive DNA damage, and depending on its severity, checkpoints activate apoptosis or senescence instead of cell‐cycle resumption. Thus, aberrant CDK activity exerts selective pressure on the checkpoints, eventually breaching them, allowing DNA damage accumulation and the emergence of GI with pathological consequences 29, 109, making CDKs attractive targets for inhibition 108. As another example, RT* is a highly regulated programme giving rise to early, middle and late time zones, and is strictly coordinated with transcription to avoid replication–transcription collision 110, 111. If this spatiotemporal process is altered by misregulation of an RT‐related gene 32 or a replication process step, e.g. replication licensing* 112, 113, the propensity to DSB formation due to replication–transcription collision increases 114, 115, 116. In this regard, low‐density replication–initiation may lead to unfinished replication, owing to the long distance between replication forks, making breakage a probable event 117. Common fragile sites (CFSs), which are late‐replicating areas of the genome with a paucity of replication origins, are particularly vulnerable to RS, and are at high risk of breaking 118. Conversely, ill‐timed replication–initiation can cause re‐replication, a form of RS that leads to DSBs and GI, and, as discussed later, is frequently observed in cancer from its earliest stages. Overproduction of replication and/or transcription intermediates that are normally formed in low amounts can also pose a threat to genome integrity (supplementary material, Figure S3A,B) 119, 120. A particular DNA:RNA hybrid termed the R‐loop* (supplementary material, Figure S3B), identified 42 years ago 121, has drawn attention as a source of GI. R‐loops usually have short lifespans (∼20 min), as they are efficiently removed under normal circumstances, and play an important role in various processes such as immunoglobulin class switches, and transcriptional regulation and termination 122. However, production of R‐loops resulting from various defects, such as lack of RBPs*, which coat and protect nascent RNA from illicit DNA hybridization 123, make these structures ‘hotspots’ for damage 124. The thermodynamically stable DNA:RNA hybrid structure of the R‐loop can impede proper completion of replication, leading to replication fork stalling and collapse*, and DSB formation, whereas the relatively unstable non‐template ssDNA of the R‐loop can act as a substrate for deaminases. Another endogenous cause of DNA damage is an inability of nucleotide biogenesis to cope with hyperproliferative states, resulting in replication fork deceleration, which can lead to fork stalling and, if not reversed, to fork collapse 125. Exogenous supplementation with nucleosides reverses these adverse effects 126.

A delicate operation that the cell has to carry out following stalling or collapse replication forks is repair and replication restart (supplementary material, Figure S3C) 127. Depending on the duration (how long) of the replication block, there are two options. (1) In short replication blocks (2–4 h), restart is mediated by fork remodelling of replication intermediate structures (fork reversal: ‘chicken foot’). Replication fork reversal is a mechanism in which a three‐way junction at a replication fork is converted to a four‐way junction by the annealing of the two newly replicated strands into a regressed arm at the forks 128. Regressed fork restart requires restoration of the typical replication fork structure, and involves helicase (RECQ‐1*, WRN*, BLM* and DNA2 nuclease*) 128, 129 and translocase (SMARCAL1*, ZRANB3* and HLTF*) activity in the resolution of these intermediates 129. Whether these helicases recognize different structures or act on the same substrates is a matter of investigation 130, 131, 132. RAD51 recombinase has been described as an essential factor in replication fork restart 127, as RAD51‐mediated strand invasion rapidly and effectively leads to replication fork remodelling 133. As the regressed arm of a reversed replication fork resembles a one‐ended DSB, it has to be protected from cleavage. RAD51‐coated nucleofilaments protect nascent DNA from MRE11*‐mediated nucleolytic attack 134, 135. BRCA2 has been shown to be dispensable for RAD51‐mediated fork reversal 136, but is crucial for the assembly of stable protective RAD51 nucleofilaments on regressed arms 134, 135, 137, 138. MRE11‐mediated degradation has recently been shown to be one of the leading causes of sensitivity to DNA‐damaging agents in BRCA‐deficient cancers 139. Recent studies have shed light on MRE11 recruitment at regressed forks by highlighting the role of PTIP*, MLL4*, and RAD52* 136, 139. Recently, a combination of electron microscopy with DNA fibre analysis further defined the events in reversed fork resection in BRCA‐deficient tumors 140. It was shown that CtIP* initiates MRE‐mediated degradation, which is then extended by EXO1* 140. How cells cope with extensive resection at the forks and how different remodellers collaborate to catalyse fork reversal is still poorly understood. (2) In long replication blocks (>24 h) resulting in fork collapse, new origins are fired in an attempt to compensate, and repair takes place by remodelling, utilizing structure‐specific endonuclease complexes* 30, 115 that generate DSBs, promoting Rad51‐dependent homologous recombination (Figure 4). The significance of the above factors is underscored by the severity of the clinical manifestations presented in disorders such as Bloom* and Werner* syndromes, in which corresponding helicases are mutated 141, 142.

### Chromatin structure, DNA damage, and DDR/R

Remarkably, susceptibility to DNA damage is not the same across the genome (where), as chromatin compaction seems to provide a ‘shield’ against DNA insults, with heterochromatin (compact chromatin) providing significant protection against breaks, but euchromatin (open chromatin), which is a relaxed and transcriptionally active conformation, being vulnerable to damage 143. A feature of heterochromatin is its high level of enrichment in repetitive DNA sequences* and its concentration in pericentromeric and telomeric genomic regions. Given that repetitive sequences represent recombination ‘hotspots’, and are thus prone to DSBs, heterochromatin formation at these sites has evolved as a protective mechanism preventing illegitimate recombination events 144. A specialized type of compact chromatin is present at telomeres* 145, 146. Telomeres resemble staggered DSBs, and, if not protected by the shelterin complex*, will be recognized as a damaged area, eliciting a DDR with detrimental effects (see ‘Monitoring mitosis: the last checkpoint’ below) 147, 148.

However, a disadvantage of compaction is that it is refractory to repair, as demonstrated by the inability to expand γH2AX 149. Studies in yeast and mammals showed fewer γH2AX foci at DSBs in heterochromatin than in euchromatin 150. When a break occurs in euchromatin, a complex of heterochromatin‐associated proteins*, including KRAB‐associated protein 1 (KAP1), heterochromatin protein 1 (HP1), and SU(VAR)3‐9H1, assist in trimethylation of H3K9, which is a repressive histone PTM. This reaction facilitates an amplification process that spreads over many kilobases from the damage 116. Activated TIP60 (see above) binds to H3K9me3, leading to ATM activation and H4 acetylation, promoting chromatin relaxation. In turn, ATM phosphorylates KAP1, releasing it from chromatin and enhancing access to the damaged site. It appears, that following DNA damage, there is a transient shift in euchromatin, like the ‘squeezing phase of an accordion’, to increase compaction to recruit TIP60 and ATM 68. Compaction can also function as a regulatory mechanism for uncontrolled DSB‐end resection and HR, as we recently showed, via retention of KAP1 and HP1 at the damaged site by the histone chaperone SET/TAF‐Ib/I2PP2A/INHAT 151. In contrast, KAP1 undergoes localized phosphorylation by ATM in a 53BP1‐dependent manner in heterochromatin, dispersing the nucleosome remodeller* CHD3.1, leading to focal relaxation, unlike the diffused relaxation observed in euchromatin 152, 153, 154. The difference in DDR initiation between euchromatin and heterochromatin influences the choice of repair, with transcriptionally active euchromatin regions preferring HRR, and compacted chromatin favouring NHEJ 143. Another repetitive area that is influenced by chromatin compaction, and that is of immense importance for genome and proteome integrity, is composed of the ribosomal DNA (rDNA) clusters, located in the nucleolus.

### The nucleolus at the crossroad of stress response

The nucleolus, which is the largest structure in the nucleus, is responsible for rRNA production and ribosomal assembly, as reflected by its characteristic tripartite spatial organization* (supplementary material, Figure S4A) 155, 156. To cope with increased protein production, rDNA is organized into clusters of gene repeats around areas termed NORs* 157. Depending on the demands of protein synthesis, an additional regulation step occurs, which involves adjustment of the number of ‘active’ repeats and alteration of their transcriptional rates 158. RNA polymerase I transcribes rDNA into rRNA, forming the framework of the ribosome. rRNA constitutes 80% of the total cellular RNA, making rDNA the highest‐transcribed locus of the genome. Obviously, the propensity for replication–transcription collisions and R‐loop formation is increased in rDNA loci, especially under conditions of oncogene‐induced RS 159. As a ‘precautionary measure’, the RFB* (supplementary material, Figure S4A), which is an intergenic rDNA site, is recognized as a site coordinating replication and transcription 160, whereas heterochromatin associated with the rDNA clusters acts as a ‘buffer zone’ against genotoxic conditions 161, 162, 163. Regardless of cellular demands, not all rDNA sequences are transcriptionally active, and a fraction of nucleolar rDNA is always silent (supplementary material, Figure S4A). The stability of the repetitive and recombinogenic‐prone rDNA sequences requires chromatin silencing complexes 162, 164, 165. The fraction of ‘active’ versus ‘silent’ rDNA is regulated by the rDNA remodelling complexes NoRC*, eNoSC*, and NuRD*, which limit accessibility to the recombinogenic machinery 166, 167, 168, 169. Disruption of rDNA‐associated silencing proteins within the inner nuclear membrane* disturbs the nucleolus–nucleoplasm boundary, induces the formation of recombination foci, and destabilizes the repeats 170, 171. Finally, almost 70% of nucleolar proteins have functions unrelated to ribosome biogenesis, including triggering SR pathways. A characteristic example is p53 activation following nucleolar segregation (ribosomal stress*) upon DNA damage or transcription inhibition (supplementary material, Figure S4A) 172, 173, 174.

### Surveillance and maintenance of mitochondrial genome integrity

Mitochondria are the ‘energy factories’ of cells, and they also regulate other vital functions, including apoptosis. The symbiotic relationship between mitochondria and eukaryotic cells started more than a billion years ago, leading to synchronized action between the two genomes, with most proteins involved in mitochondrial DNA (mtDNA) metabolism being encoded by nuclear DNA 175. Conversely, proteins participating in oxidative phosphorylation are encoded by mtDNA, making mtDNA integrity of paramount importance for cell homeostasis 176. mtDNA is more prone to damage than nuclear DNA, with a 10–20‐fold higher mutation rate, possibly because of proximity to reactive oxygen species (ROS) production (supplementary material, Figure S4B).

mtDNA is an approximately 16‐kb closed circular DNA containing a specific regulatory region, termed the D‐loop*, harbouring initiation sites for both replication and transcription. The mitochondrion‐specific polymerase‐γ (pol‐γ) holoenzyme is responsible for replication, with >200 mutations in *POLG* having been linked to mitochondrial diseases 177. In contrast to the nuclear genome, which is organized into nucleosomes, mtDNA lacks nucleosomes and is arranged as protein–DNA complexes known as nucleoids. Interestingly, super‐resolution microscopy has revealed that the mtDNA packaging density is higher than that of nuclear chromatin, forming a ‘shield’ against mutagenesis 178. Interestingly, Twinkle helicase is essential for mtDNA maintenance and a key regulator of mtDNA copy number by linking the mitochondrial replication machinery with the cytoplasmic dNTP pool 179. As mitochondria are the largest consumers of dNTPs in the cell, controlling mtDNA copy number is apparently essential for meeting cellular energy requirements 180.

Oxidative damage is a common mechanism of mitochondrial injury. Byproducts of oxidative phosphorylation are ROS, because, during the series of redox reactions, a small percentage of electrons leak directly to oxygen. Thus, 1–2% of the oxygen consumed within the cell is released from mitochondria as ROS. Under normal conditions, the amount of ROS produced is relatively low and essential for proper intracellular signalling, metabolism, and responses to pathogens; however, during periods of increased and/or prolonged ROS production, extensive and persistent mtDNA damage may occur (supplementary material, Figure S4B) 181. BER is the predominant DNA repair pathway for category‐S lesions in mitochondria, whereas they lack effective MMR and are deficient in NER. Adducts requiring NER for their removal will accumulate, resulting in mtDNA mutations and, ultimately, mtDNA degradation 182. Importantly, pol‐γ can bypass some lesions by TLS. Regarding TLS, primase‐polymerase (PrimPol), which is an archaic enzyme with dual primase and polymerase activities, identified in human mitochondria, has the unique feature of *de novo* DNA synthesis, and the ability to tolerate lesions such as 8‐oxoguanine (8‐oxoG) and apurinic/apyrimidinic or abasic (AP) sites 183. Along the same line, Pif1, a 5′–3′ DNA helicase that is essential for mtDNA replication 184, is involved in the repair of many types of mtDNA damage, including the unwinding of genotoxic G‐quadruplex DNA* 183. For category‐D lesions, the prevailing view is that both HR and NHEJ are active in mitochondria, although recent evidence suggests that NHEJ is replaced by microhomology‐mediated end‐joining (MMEJ), also known as alternative non‐homologous end joining (alt‐NHEJ) (Figure 4) 185.

Depending on the type and amount of damage, inefficient mtDNA repair activates either mitochondrial fusion* or fission* 186. Fusion rearranges the matrix content of a damaged mitochondrion with a healthy one; this event results in diluting the damage that relates e.g. to unfolded proteome or to mutated DNA. On the other hand, fission partitions damaged material to daughter organelles. If the above fail, mitophagy* or apoptosis will take place, determined by the extent of damage (supplementary material, Figure S4B) 187. Molecular players dictating the outcome include ATM, p53, and Sirt1* 188. Sirt1 is a master regulator inhibiting p53‐mediated apoptosis, and, by interacting with AMPK*, directs the SR to mitophagy. Concurrently, by deacetylating PGC1‐a*, it stimulates mitochondrial biogenesis to compensate for losses.

### Monitoring mitosis: the last checkpoint

The cell's final level of surveillance occurs during mitosis, as its genetic material must be accurately and equally transferred to offspring. Mitosis is an orchestrated process leading to aneuploidy, CIN* or death if deregulated (supplementary material, Figure S5) 189, 190, 191. Owing to its complexity and short duration (∼1 h), multiple control checkpoints ensure the fidelity of genome inheritance 192, 193. Identified checkpoints occur: (1) between prophase and prometaphase, controlled by checkpoint with forkhead and ring finger domain (CHFR), which is implicated in sensing microtubule poisons 194, 195; (2) during metaphase, governed by the SAC* 196; and (3) in telophase, regulated by the cytokinesis or ‘abscission’ checkpoint* (AC), which is Aurora‐B*‐dependent (supplementary material, Figure S5) 197, 198, 199.

The SAC is the most important surveillance mechanism, monitoring kinetochore–microtubule attachment, and ensuring correct alignment of chromosome pairs before segregation 200, 201, 202. During metaphase, the unattached kinetochores recruit the SAC machinery*, which sequesters CDC20* to block activation of the APC/C* 203. Proper chromosome alignment results in SAC silencing, allowing activation of APC/C, which, in turn, targets securin* and cyclin‐B* for degradation 204. Destruction of securin releases separase*, which cleaves the cohesin rings*, promoting sister chromatid separation (supplementary material, Figure S5). The significance of SAC was demonstrated in mouse models showing that complete loss of these genes results in early embryonic lethality, whereas heterozygous and hypomorphic* mice are viable and fertile despite showing increased levels of aneuploidy. Although aneuploidy is evident in these mice, malignant transformation is a rare and late event 189, questioning whether aneuploidy as such is causative for cancer development 205. Differences in cancer incidence among individuals with identical aneuploidy, as well as between common genetic disorders such as trisomies 13 206, 18 207 and 21 208, suggest that additional hits are required for cancer to evolve. Moreover, extensive analysis in common sporadic cancers with aneuploidy demonstrated the low frequency of mutations in caretaker genes*, including SAC genes, arguing against their role in promoting cancer 29. In this regard, the role of centrosome* aberrations in aneuploidy and CIN 189, 209, 210 requires more clarification, especially in view of emerging concepts such as ‘centrosome inactivation checkpoint’ 211 that link components of the DDR/R machinery with centrosome ‘status’ and ‘mitotic catastrophe’* (supplementary material, Figure S5) 211, 212. Likewise, how delayed abscission affects chromosome homeostasis requires further investigation 199. Unexpectedly, overexpression of SAC genes is a more frequent event in human cancer 29. Conditional Mad2 upregulation predisposes mice to a wide range of early‐onset, aneuploid malignant tumors 213. The main difference from haplosufficienct *Mad2* mice, which develop only benign tumors 189, 214, is the occurrence of extensive structural aberrations, implying that the structural branch of CIN, involving DSBs, lies behind cancer progression 109, 215, 216 (see the next section). Notably, p53 and pRb inactivation lead to Mad2 overexpression, CIN, and a malignant phenotype encompassing highly aggressive features 217, 218, 219. These data corroborate previous reports linking p53/pRb expression aberrations with aneuploidy and CIN in common human malignancies 220, 221. Overall, these observations support the idea that deregulation of critical checkpoints impinges on mitotic surveillance and fidelity, determining the fate of daughter cells.

The significance of engaging the repair machinery in the correct context (where and/or when) is vital. During interphase, DNA repair is essential to maintain genome stability, whereas in mitosis it may be deleterious 222. Induction of DSBs in mitosis leads to a ‘muted’, non‐classic DDR (supplementary material, Figure S5). Mitotic DSBs are marked by MRN, MDC1 and γH2AX foci in a PI3K‐dependent manner, whereas RNF8 and RNF168 localization with mitotic γH2AX does not take place, excluding the recruitment of 53BP1 and BRCA1 223, thus blocking NHEJ and HRR, respectively 224. By the suppression of DRR/R in mitosis, telomere–telomere fusion is avoided. In metaphase, the shelterin complex aquires a loose configuration, termed telomere dispersion, promoting condensin loading and chromosome segregation 225, that concurrently renders chromosomes prone to fusions favouring structural CIN 222. Notably, restoration of RNF8 and 53BP1 accumulation at mitotic DSB sites results in telomere–telomere end‐to‐end fusion and aneuploidy, especially in the presence of exogenous genotoxic stress 226. Likewise, mitotic DDR/R activation can lead to deleterious chromosomal alterations 224. In particular, instead of suppressing Plk1*, as it does in G_2_ phase 227, 228, 229, it upregulates Plk1 activity and, along with Aurora‐A*, it increases kinetochore*–microtubule stability, favouring merotelic attachments*, lagging chromosomes*, micronucleus formation*, and chromothripsis* (supplementary material, Figure S5) 230, 231, 232, 233.

The outcome of mitotic DDR/R activation depends on the underlying stress parameters (Figure 1B). For instance, cells with DNA damage but intact p53 (cellular context) escape or slip out from mitotic arrest and succumb to G_1_ arrest, whereas p53‐deficient cells continue to cycle and become aneuploid 234, 235. Likewise, in cells with low DNA damage levels (how much), mitotic DDR/R marks DSBs (memory signals) for repair in the subsequent G_1_ phase 236, 237, 238, resulting in a similar fate to that of cells with DNA damage and signalling effects occurring in earlier cell‐cycle phases 239, 240, 241. In contrast, excessive DNA damage (how much) activates the SAC, leading to mitotic arrest 237. Arrested cells may either die by mitotic catastrophe* or may exit mitosis prematurely without proper chromosome segregation and cytokinesis, through a process termed mitotic slippage* (supplementary material, Figure S5) 242. The destiny of these cells is not clear. They can follow the road to cancer, acquire a senescent phenotype, or die (supplementary material, Figure S5). Altogether, rewiring DSB repair to a ‘repressive mode’ during mitosis has advantages and disadvantages. Specifically, as execution of classic DDR/R is an ATP‐consuming process, it possibly saves energy for the demanding process of mitosis 243; also, it favours rapid execution of mitosis at the expense of increased sensitivity to DNA‐damaging stressors; and it protects against structural alterations. On the other hand it is prone to causing numerical CIN.

## Overview of the proteostasis network (PN): connecting the PDR with DDR

Downstream of the critical, but ‘lifeless’, genetic information, there is a world of immense complexity, namely the proteome. The entry point to this world occurs via protein synthesis, which takes place in the cytosol and endoplasmic reticulum (ER) by a complicated machine (i.e. the ribosome) that (1) decodes the information stored in nucleic acids, and (2) shifts the chemistry from nucleic acids to amino acids.

### Overview of the PN: links with DDR/R

Human cells express millions of polypeptides of >10 000 different species 244 that fold into well‐defined three‐dimensional structures that form parts of protein machines. Because the average protein and proteome sizes have increased significantly during evolution 245, and the consequences of an unstable proteome can be catastrophic 246, 247, cells have evolved a modular, but integrated, system that ensures general proteome quality control, called the PN 248. The PN performs the daunting task of curating polypeptide synthesis, folding, conformational maintenance, sorting–trafficking, and degradation; it also responds to conditions of proteotoxic stress by addressing the triage decision of fold, hold, or degrade (supplementary material, Figure S6A). The PN comprises ∼2000 chaperones, folding enzymes, trafficking modules, and degradation components, and it is not surprising that proteome stability maintenance consumes the majority of cellular ATP 249, 250. Because of the wiring and interdependence of various PN branches, defects in one module trigger a breakdown of the entire network; these effects are evident during ageing and in age‐related diseases such as cancer 251.

The ‘chaperome’ consists of hundreds of cytosolic and organelle‐specific chaperones that, along with their associated factors, bind to a wide range of distinct substrates 252, 253. Chaperones* are involved in proper polypeptide folding, unfolding, and remodelling, and in the assembly of protein machines or the delivery of damaged polypeptides to degradation machineries (supplementary material, Figure S6). Nuclear chaperones provide examples demonstrating how components of the PN assist the DDR/R machinery, unveiling a new role of proteome quality control in preserving genomic stability (supplementary material, Figure S6A). Specifically, nuclear chaperones orchestrate the delivery of newly produced histones to DNA, and also facilitate histone turnover 68, 254. Hence, reassembly of chromatin upon DNA repair is highly dependent on chaperones 51, 255.

When folding of a mutated or a post‐translationally irreversibly modified polypeptide fails, cellular proteases take over. The two main branches of this part of the PN are the UPP and autophagy–lysosome (ALP) pathways, comprising ∼850 and ∼500 different components, respectively (supplementary material, Figure S6A) 256, 257, 258, 259, 260. The UPP pathway degrades short‐lived poly‐ubiquitinated normal proteins and non‐functional or misfolded polypeptides and is composed of ubiquitin‐conjugating enzymes and the 26S (or 20S) proteasome* 261, 262. The ubiquitin ligase family confers substrate specificity and comprises almost 600 genes in humans 257, 263. The UPP pathway is central to protein synthesis quality control, as non‐functional newly synthesized polypeptides are targeted for degradation to cytosolic or ER‐bound proteasomes [ER‐associated protein degradation (ERAD)] 264. Proteasomes are also located in the outer mitochondrial membrane, and perform outer mitochondrial membrane‐associated degradation (OMMAD) during activation of the mitochondrial unfolded protein response (UPR^MT^) 265. The UPP pathway is also involved in the degradation of mitochondrial fusion/fission proteins, and thus, apart from genome and proteome stability, PN modules are also critical for mitostasis* 186. It is not thus surprising that ubiquitin ligases are frequently deregulated in cancer 262.

The ALP pathway is an intracellular self‐catabolic process that comprises three forms in mammalian cells: (1) chaperone‐mediated autophagy (CMA); (2) microautophagy; and (3) macroautophagy 12, 266, 267, 268. In CMA, damaged polypeptides are degraded in lysosomes after being recognized by chaperones, which bind to the lysosomal LAMP2A receptor 269; CMA offers an alternative to the UPP pathway for degradation of misfolded proteins. Microautophagy is a less well understood process involving engulfment of small cytosolic regions by the lysosomal membrane. Finally, in macroautophagy, the Atg* proteins form autophagosomes that capture lipids, proteins, or even organelles, transferring them to lysosomes for degradation 270. The ALP pathway also degrades ubiquitinated substrates (including protein aggregates) via the action of microtubule‐associated histone deacetylase 6 (HDAC6) and sequestrome‐1 (p62/SQSTM1) 271.

Genome damage results in the activation of proteostatic modules, and crosstalk between the DDR/R machinery and autophagy has been established (supplementary material, Figure S6A) 272, 273, 274. Prominent connections include those between ATM and PARP1 with AMPK*, one of the central metabolic regulators in eukaryotes. AMPK is activated when the AMP/ATP ratio is high, setting in motion autophagic flux 275, 276, 277. Likewise, p53 has been shown to upregulate components of autophagy, thus forming an amplification loop 278. Autophagy modulates DNA repair by degrading (among others) KAP1, HP1, and sequestrome‐1 (p62/SQSTM1), which hinder BRCA1 and Rad51 accessibility to DSB sites, promoting successful completion of HRR 279. Similarly, the UPP pathway and ubiquitylation are integral parts of the DDR pathway, as ubiquitylation of DDR factors has emerged as a switch that initiates signalling cascades and also as a proteolytic signal coordinating recruitment and disassembly of these proteins 280. Furthermore, the proteasome is involved in the degradation of DNA repair proteins following completion of the process (see above). Given the extensive ongoing damage and genome remodelling during cancer, it is not surprising that proteostatic machineries are deregulated during oncogenic transformation (supplementary material, Figure S6B) 251, 262, 281. However, the link between DDR/R and the PN or the PDR warrants further investigation, especially during carcinogenesis (supplementary material, Figure S6C), as this interrelationship is not straightforward. Specifically, recent *in vivo* data indicate an inverse relationship in precancerous lesions, whereas, at the cancerous stage, PN modules and the DDR operate in parallel 272, 274, 281.

### PDR signalling

Polypeptides are post‐translationally modified either by PTMs, which are normal regulatory processes and do not increase proteome instability, or by non‐enzymatic protein modifications (NEPMs), which are stochastic and disrupt their structure and function 282. Unfolded or damaged proteome components impinge on the PN, triggering a response pathway called PDR (see Introduction) (supplementary material, Figure S6A). The PDR branches are coordinated by a series of complementary homeostatic mechanisms, which sense and respond to imbalances in proteostasis and/or to increased amounts of stressors. These signalling cascades, namely the heat shock response, hypoxia response, oxidative stress response, unfolded protein response in the ER (UPR^ER^)*, and UPR^MT^*, are modulated by transcription factors that sense stress and mobilize genomic–cytoprotective responses 274, 281, 283. These responses are also coupled with a decrease in protein synthesis, thereby reducing the influx of newly synthesized proteins and/or allowing preferential translation of stress‐responsive mRNAs until proteome stability restoration 284, 285, 286.

Proteome instability is mainly counteracted by heat shock factor 1 (Hsf1), which is maintained in an inert state by chaperone binding 287. Upon heat or other types of stress that destabilize the proteome, these chaperones are titrated away from Hsf1 by binding to denatured proteins, and Hsf1 thus translocates to the nucleus, inducing transcription of a wide range of proteostatic modules 288, 289. Similarly, chaperones that are localized in the ER guide the folding of membrane or secreted proteins. Furthermore, the ER has its own stress response pathway (UPR^ER^) that is activated in cases of increased flux or heavy secretory loads, or after heat shock that increases protein misfolding 290. The UPR^ER^ attenuates *de novo* protein synthesis and induces the expression of chaperones to aid proper polypeptide folding, or, if organelle proteostasis cannot be restored, triggers apoptosis. Likewise, the UPR^MT^ maintains mitochondrial functional integrity by increasing the rates of polypeptide folding and degradation through the transcriptional activation of specific mitochondrial chaperones and proteases 291, 292, 293. Additionally, the UPR^MT^ induces OMMAD, mitophagy, or apoptosis if disruption of mitostasis is irreversible 293, 294. An SR of paramount importance for cellular survival is triggered by hypoxia and is regulated by the Hif‐1 transcription factor 295. Under normoxic conditions, Hif‐1 is targeted for proteosomal degradation by the VHL E3‐ubiquitin ligase. In hypoxic conditions, Hif‐1 is protected, activating a transcriptional programme that includes (among other things) the upregulation of chaperones 296. Finally, the Nrf2 signalling pathway plays a crucial role in defence against oxidative and/or xenobiotic damage by activating [after binding to antioxidant response elements (AREs)] a broad range of detoxification enzymes 297, 298 and by inducing UPP and ALP pathway genes 299, 300.

### The PDR and DDR/R: an intermingled fate

Unmitigated stress will eventually exceed the buffering capacity of the PN, leading to proteome instability (supplementary material, Figure S6B) 301, which is a daunting prospect. As the DDR/R machinery includes complex protein machines, the faithful execution of the triage ‘access–repair–restore’ is obviously dependent on proteome stability. Failure of the PDR pathway to cope with proteotoxic stress poses a risk for genomic integrity. Conversely, constitutive DDR/R activation will eventually wear out the proteome because of defective transcription and reduced polypeptide quality (supplementary material, Figure S6B). Suppressed ribosomal biogenesis during DNA damage is an example of how activation of DDR/R may affect proteostasis 302. Furthermore, sustained DNA damage can compromise DDR/R efficiency, increasing the production of mutated polypeptides and proteome instability. Subsequently, a vicious cycle of low‐fidelity DDR/R–PN activity develops that will trigger pathophysiological states, including carcinogenesis 303. The most characteristic paradigm of a ‘saturated’ PDR linked with aberrant DDR activation is accumulation of lipofuscin during oncogene‐induced senescence, with detrimental effects for the cell (see the next section; supplementary material, Figure S6).

## Oncogene‐induced DNA damage and cancer development; ‘a model to rule them all?’

In a seminal paper in the early 1990s, Fearon and Vogelstein 304 proposed a genetic model for cancer development, using colorectal cancer as the basis of their study. They suggested that salient molecular alterations characterize each morphological stage of colorectal cancer progression, from the early small adenomas (benign phase) to the large metastatic carcinomas (malignant phase). Molecular changes increase from the benign to the malignant phase, and include mutational activation of oncogenes* coupled with inactivation of tumor suppressors* 305. Since then, the general features of this model have been applied to other epithelial neoplasms 306. The idea of analysing the molecular traits of each developmental stage of cancer has revolutionized the field and played a major role in the emergence of molecular pathology. Twenty years later, findings largely based on the above models led Hanahan and Weinberg 307 to propose six hallmarks of cancer. These include sustained proliferative signals, inactivation of tumor suppressors, resistance to apoptosis, replicative immortality, increased angiogenesis, and invasion/metastasis. Conceptual advances in the last decade provided two additional hallmarks: escape from immune surveillance and deregulated metabolism 308.

In spite of the value that the above model provided by setting the timeline of molecular events, it does not offer an explanation of how one molecular alteration leads to the next. In other words, it is a static model lacking the dynamic parameter of the driving force. Some years ago, we put forward a model for cancer development postulating that oncogene‐induced DNA damage followed by error‐prone repair could be the link between the hallmarks of cancer 109. The concept was based on the simple finding that carcinomas show DDR foci from their earliest stages of development, whereas adjacent normal epithelium is ‘clear’ 309, 310. This observation suggested that an endogenous source within the incipient cancerous environment caused DNA damage, triggering constitutive activation of the DDR pathway. We hypothesized that a hyperproliferative state could be the origin. Hyperproliferative signals, in most cases, represent parts of SR pathways that deviate from their adaptive/protective role, particularly when SR parameters stray beyond certain limits. For example, squamous metaplasia of the bronchial epithelium is an adaptive response to toxic injury caused by cigarette smoke stimulated by the epidermal growth factor receptor (EGFR) pathway 311. Depending on the cellular and environmental context, squamous metaplasia can shift progressively to dysplasia and to full neoplastic transformation in which *EGFR* becomes frequently amplified, fulfilling the first hallmark of cancer [Figure 5(1), lower panel] 307, 312, 313.

To functionally recapitulate the above scenario, we utilized various types of normal cells and precancerous models, and showed that hyperproliferative stimuli, including activated oncogenes, caused DSBs 159, 309, 310, 314. Interestingly, the lesions occurred predominantly at CFSs* 159, 309, 314, 315. As mentioned above, CFSs are late‐replicating regions of the genome with a sequence composition that makes them vulnerable to RS [Figure 5(2,3); lower panel] 118, 316, 317. As a result, they show breaks, losses and rearrangements, which are genetic alterations collectively termed CFS expression [Figure 5(3); lower panel] 118, 318, 319, 320. Their expression is not just a ‘passive’ event, but an incident with severe repercussions, as CFSs constitute highly ‘functional’ entities harbouring cancer‐related genes, often extending over large genomic regions (long genes), microRNAs (miRNAs), and regulatory sequences, with a much higher density than in non‐fragile sites 118, 321. For instance, two of the long genes located within CFSs are *FHIT* in FRA3B, and *WWOX* in FRA16D, both of which are potent tumor suppressors that are frequently inactivated in cancer 322, 323, 324, 325. Consistently, sequencing of colon adenomas revealed that SNSs mapped more often to very large genes 326. Likewise, in a precancerous cellular model, we noticed that, apart from CFSs, the repetitive and damage‐prone rDNA (see ‘The nucleolus at the crossroad of stress response’ above) was an additional ‘hotspot’ for oncogene‐mediated damage [Figure 5(3); lower panel] 159.

The above observations led us to suggest that activated oncogenes compromise DNA replication, provoking RS and, in turn, activating the DDR/R pathway in an effort to ‘fix’ the generated lesions [Figure 5(2); lower panel]. Concurrently, to impede the transition of mutated genetic material to offspring, the DDR sets in motion the antitumor barriers of apoptosis and senescence 109, 159, 309, 310, 314, 327, 328, 329, 330. However, sustained RS would lead to accumulation of damage that, at some point, would overwhelm the capacity of the high‐fidelity DNA repair routes, shifting error‐free to error‐prone repair [Figure 5(2,3)]. This switch will, in due course, modify the genome landscape, exhausting the integrity of antitumor responses and paving the way for cancer progression [Figure 5(2–5); lower panel].

A prediction of this model whereby oncogene‐induced RS (OIRS) acts as a driving force for cancer progression is that the replication machinery and its regulatory network* should play a vital role in cancer initiation and progression [Figure 5(2); lower panel] 331, 332. Genes that either positively or negatively regulate growth would be primary targets for activating or inactivating mutations, respectively, whereas DNA repair genes will be spared to ‘fix’, as otherwise the incipient cancer cell will die. Recent high‐throughput sequencing studies in sporadic cancers support this notion, showing: (1) a high frequency of inactivating mutations in checkpoint genes such as *RB* (retinoblastoma), *TP53*, and *ATM*; (2) a high incidence of activating events (e.g. mutations and gene amplifications) in growth‐promoting genes such as *RAS* and *EGFR*; and (3) a paucity of mutations in DNA repair genes 29. Particular attention should be given to the p16^INK4A^–Rb–E2F pathway, which is a major ‘molecular crossroad’ that most mitogenic/oncogenic signals converge to. Its phosphorylation status determines whether the cell will bypass the ‘restriction point’ of G_1_ phase, committing the cell to progress to S phase without the requirement for extracellular stimulants 112, 113, 114, 115, 116, 117, 118, 119, 120, 121, 122, 123, 124, 125, 126, 127, 128, 129, 130, 131, 132, 133, 134, 135, 136, 137, 138, 139, 140, 141, 142, 143, 144, 145, 146, 147, 148, 149, 150, 151, 152, 153, 154, 155, 156, 157, 158, 159, 160, 161, 162, 163, 164, 165, 166, 167, 168, 169, 170, 171, 172, 173, 174, 175, 176, 177, 178, 179, 180, 181, 182, 183, 184, 185, 186, 187, 188, 189, 190, 191, 192, 193, 194, 195, 196, 197, 198, 199, 200, 201, 202, 203, 204, 205, 206, 207, 208, 209, 210, 211, 212, 213, 214, 215, 216, 217, 218, 219, 220, 221, 222, 223, 224, 225, 226, 227, 228, 229, 230, 231, 232, 233, 234, 235, 236, 237, 238, 239, 240, 241, 242, 243, 244, 245, 246, 247, 248, 249, 250, 251, 252, 253, 254, 255, 256, 257, 258, 259, 260, 261, 262, 263, 264, 265, 266, 267, 268, 269, 270, 271, 272, 273, 274, 275, 276, 277, 278, 279, 280, 281, 282, 283, 284, 285, 286, 287, 288, 289, 290, 291, 292, 293, 294, 295, 296, 297, 298, 299, 300, 301, 302, 303, 304, 305, 306, 307, 308, 309, 310, 311, 312, 313, 314, 315, 316, 317, 318, 319, 320, 321, 322, 323, 324, 325, 326, 327, 328, 329, 330, 331, 332, 333. Hence, inactivation of p16^INK4A^–Rb signalling is not a surprising finding in cancer 221, 334, 335, 336, 337, as it releases E2F transcription factor 1 (E2F1) from its inhibitory control 329, 333, 338, 339, stimulating the sustained expression of cell‐cycle drivers such as cyclin E, Cdc6, and Cdt1, converting them into ‘oncogenic stimuli’ [Figure 5(1); lower panel] 112, 210, 314, 327, 338, 340. In support of this, we observed that E2F1, Cdc6 and Cdt1 are aberrantly expressed in most cancer types examined, and, importantly, from their earliest stages 112, 314, 329, 338, 339, 340. Deregulated production of E2F1, Cdc6 and Cdt1 was not a mere reflection of an increased proliferation rate, as their forced expression (utilizing various inducible cellular systems covering the whole spectrum of carcinogenesis) triggered RS, DNA damage and DDR activation with induction of apoptosis and/or senescence 159, 314, 327, 329, 340, 341, 342. In accordance with the main prediction of our model, p53 function was gradually attenuated, apoptosis was reduced, and a fraction of senescent cells escaped, showing aggressive traits [Figure 5(4–6); lower panel] 159, 314, 327, 342, such as increased invasiveness, aneuploidy,* and features reminiscent of EMT* 159, 314, 343, which is an embryonic developmental programme exploited by cancer cells to invade and metastasize 344, 345. The above sequence of events explains why p53 needs to be inactived in tumors for oncogenes to exert their adverse effects 221, 335, 338, 339, 346, 347, 348, 349, 350.

The functional coupling between oncogene activation and inactivation of tumor barriers provides experimental evidence that OIRS acts as a driving force exerting selective pressure that eventually shapes the stage for cancer progression (Figure 5). The mechanism behind OIRS and cancer development depends on the availability of ‘quantitative and qualitative repair resources’. When the latter condition is not met, continuous rewiring of DNA repair networks occurs, favouring the survival of the ‘fittest incipient cancer cell’. As a consequence, chronic activation of the DDR/R network results in the exhaustion of essential short‐lived repair factors controlling high‐fidelity repair, such as Rad51 or 53BP1, thus forcing repair to follow less accurate routes 342, 351, 352. Within this framework, we noticed that, in a p53‐deficient environment, p21^WAF1/Cip1^ (a traditional tumor suppressor) revealed a ‘dark side’ of promoting GI by deregulating the replication licensing machinery and rewiring the DNA repair network to favour Rad52‐dependent error‐prone break‐induced replication (BIR) and single‐strand annealing (SSA) [Figure 5(2,3); lower panel] 342, 353, 354. This observation challenges the conventional view of dividing cancer‐related genes into ‘oncogenes’ and ‘tumor suppressors’, stressing the significance of cellular context in protein function.

As the activities of the DDR/R network go well beyond the boundaries of just ‘sensing, signalling and repairing DNA lesions’, by crosstalking with crucial SR pathways, OIRS‐mediated DNA damage makes the situation more complex 23, 274, 355. It is thus apparent that, when DDR components such as p53 and ATM are targeted, essential intercellular and intracellular surveillance operations will be modified, favouring, in the end, cellular transformation. There are two additional DDR interactions worth mentioning: (1) the immune system; and (2) metabolic pathways [Figure 5(3); lower panel]. Regarding the first, the DDR pathway communicates with nuclear factor‐κB (NF‐κB), the central hub of the immune response. Two of the best‐characterized connections involve ATM‐dependent activation of NEMO, a regulatory subunit of the IKK complex, stimulating NF‐κB activity 356, 357, 358, 359, 360, and of NKG2D and DNAM‐1/CD226 ligands, two key players in innate imunity 361, 362, 363. Another paradigm of DDR and immune response crosstalk is that between p53 and *ICAM1* 364, 365, 366, showing a direct role of p53 in immunosurveillance. As a result, tumor‐promoting inflammation, which is an enabling characteristic of cancer 308, can be attributed, to a certain degree, to a sustained DDR 23, 109, 367, 368, 369. Considering the link with metabolism, the most prominent example is that of p53 inhibiting glycolysis by inducing TIGAR 355, 370. Within this context, cells deficient in ATM or p53 will create, over time, a permissive environment favouring immune evasion and ‘aerobic glycolysis’, both of which are hallmarks of cancer cells (Figure 5).

Collectively, the above data further support the role of GI in carcinogenesis, making it a hallmark of cancer [Figure 5(3); lower panel] 29, 308. Nevertheless, although our model provides a unified mechanistic explanation of how the hallmarks of cancer are formulated during cancer development, several questions remain to be answered (Figure 5). For instance, how does OIRS lead to replicative immortality? Surprisingly, reactivation of telomerase, which occurs in almost 90% of human cancers, is attributable to two nucleotide substitutions, C228T and C250T, located within the *TERT* promoter, implying that error‐prone repair was involved in the selection of these mutations (Figure 5) 371. Another issue is how angiogenesis is induced within the context that we propose. A potential mechanism could be via ROS production, which prevents Hif‐prolyl hydroxylase from activating Hif, which, in turn, stimulates neovascularization 372. Escaping senescence is an emerging concept in cancer progression. OIRS seems to play a role in shaping the genetic landscape for ‘escape’ to occur 159, 342, 354, 373. However, apart from genetic alterations, epigenetic modifications, extensive chromatin remodelling, increased proteome instability and metabolic reprogramming should also take place for such a dramatic shift in cellular behaviour to occur 68, 251, 374, 375, 376, 377. Addressing the mechanistic details linking OIRS with these molecular adjustments would help immensely in finding the ‘Achilles’ heel' and designing analogous therapeutic strategies.

## Therapeutic strategies, novel tools, and future perspectives

If OIRS and error‐prone repair drives cancer, targeting this process will kill cancer cells 303, 378, 379. This hypothesis has been tested by designing various relevant therapeutic approaches over the last few years. The most characteristic example is PARP inhibition. In 2014, olaparib was the first PARP inhibitor approved for the treatment of advanced *BRCA1*/*2* mutant ovarian cancer 380, 381. ATR or Chk1 inhibitors are not particularly toxic for normal cells, but cancer cells harbouring DNA lesions rely on ATR for survival. Two highly selective and potent ATR inhibitors, AZD6738 and VX‐970, are in early‐phase clinical trials, either as monotherapies or in combination with a variety of genotoxic chemotherapies 382. In addition to inhibitors of RS, cell‐cycle checkpoint inhibitors against the Wee1 kinase and several CDKs are also under development as stand‐alone or combination therapies 383, 384. A parameter to be taken into consideration in future therapeutic interventions is the circadian rhythm, as increasing evidence has demonstrated its impact on key biochemical functions, including the DDR/R 385, 386, 387, 388. This constraint should be into consideration when novel therapeutic modalities are designed, to augment the therapeutic effect.

The dramatic changes that occur in cellular metabolism during cancer development have drawn attention to novel therapeutic approaches. Specifically, the increased oxidative stress that characterizes most cancer cells has been an object of intense investigation. Targeting enzymes that hydrolyse and remove oxidized nucleotides, such as mutT homologue 1 (MTH1), has shown promising results 389, 390, 391, 392. An additional relationship that can be exploited therapeutically is that between ATM and alternative reading frame (ARF). ARF is encoded together with p16^INK4A^ by the *CDKN2* locus. Historically, it was the first tumor suppressor reported to sense and react to oncogenic stimuli 393. It is interesting that, for many years, it was considered to act in a DDR‐independent manner, showing multiple functions such as regulating p53 stability and ribosome biogenesis 394, 395. We recently showed that ATM keeps ARF in check, as a ‘second line of defence’ 174. Moreover, ARF shows a higher activation threshold to ‘oncogenic load’ than the DDR pathway, thus forming together the DDR with a hierarchically organized ‘antitumor barrier’ 330. Consequently, targeting ATM, particularly in p53‐deficient tumors, will probably set in motion the antitumor properties of ARF, simultaneously crippling DDR signalling and cumulatively inhibiting tumor growth 174.

As the functionality of proteostatic modules 261 and anti‐stress responses 298 decline during ageing, these events also fuel ageing and age‐related diseases, including cancer (supplementary material, Figure S6B,C) 248, 301, 396. However, during the late phases of carcinogenesis and because of accumulating stressors, there should be a selective pressure for upregulation of the cytoprotective PN, and advanced and/or metastatic tumors may thus become ‘addicted’ to the higher expression levels and/or activities of proteostatic modules [Figure 5(3); lower part]. This hallmark of advanced tumors can be exploited therapeutically, because, apart from the increased load of mutated polypeptides, the high replication rates of cancer cells requires upregulated protein synthesis and maintenance that are out of balance relative to differentiated cells. Indeed, cancer cells are highly sensitive to Hsp90 inhibitors 397, and proteasome inhibitors have shown clinical efficacy in haematological cancers 398. Likewise, the ‘stress phenotype’ of cancer cells offers novel therapeutic strategies, as cancer cells have probably exhausted their capacity to survive under conditions of increased stress. In support of this, it was found recently that piperlongumine (a compound that upregulates ROS in both cancer and normal cells) had selective antitumor effects with no apparent toxicity in physiological tissues 399.

However, one thing that cancer has taught us is its resilience, adaptation to therapy, and evolution 400. One of the most intriguing features of cancer evolution, aside from loss or enhancement of function, is gain of novel functions, further increasing the level of ‘plasticity’. Evasion from senescence, circumvention of immune surveillance and p53 ‘gain‐of‐function’ mutations are representative examples 159, 342, 349, 373, 401, 402, 403, 404, 405, 406, 407. A decade ago, the weapons in our arsenal with which to investigate such complicated phenomena were still limited. The era of ‐omics provides us with tremendous amounts of data, and molecular resolution capabilities have reached the single‐cell level 408. Nevertheless, the rapid increase in the amount of information can lead to erroneous or misleading conclusions in certain cases, challenging established knowledge. At this point, advanced bioinformatics tools combined with sophisticated molecular methods take centre stage, unveiling hidden patterns and providing accurate mechanistic insights into disease and particularly cancer development 409, 410, 411. For instance, whole genome sequencing, even at the single‐cell level, can identify mutational signatures that actually represent the repair history of a cancer cell, thus highlighting potential therapeutic targets 27, 28, 39, 412, 413. Other revolutionary methods include chromosome conformation capture techniques that facilitate a three‐dimensional view of genome–proteome interactions, providing, for the first time, unique opportunities to monitor ‘holistically’ the regulation and deregulation of homeostatic mechanisms 414, 415, 416. Nevertheless, conventional methods still play a role, not only at the diagnostic level but also at the front line of research. The best example is the emergence of lipofuscin, a substance identified 175 years ago by Adolf Hannover, as a key hallmark of cellular senescence (supplementary material, Figure S6D) 417, 418. On the basis of this feature, we recently developed a novel reagent that is able to monitor senescence in any biological setting, including archival material, providing a solution to a challenge that haunted the field for almost 50 years 418. The latter is of vast importance now that senolytic drugs are entering the scene. From all of the above, it is evident that we are moving from the ‘era of ‐omics’ to the ‘era of quantum bioinformatics*’ 419; thus, for the first time, the prospects for precision medicine are bright.

## Author contributions statement

VGG and IPT conducted writing and manuscript preparation. DEP and ISP performed the literature search, and figure and manuscript preparation. VGG supervised manuscript preparation.

**SUPPLEMENTARY MATERIAL ONLINE**

**Supplementary figure legends**

**Full legends for main figures**

**Glossary and list of abbreviations**

**Figure S1.** The cellular fate following genotoxic insults

**Figure S2.** Other pathways that contribute to DDR signaling

**Figure S3.** Replication‐transcription intermediates and replication fork restart

**Figure S4.** Nucleolus and rDNA organization and maintaining mitochondrial DNA integrity

**Figure S5.** Monitoring Mitosis (DDR surveillance)

**Figure S6.** Functional interplay and interdependence of genome and proteome maintenance modules (DDR and PDR surveillance)

**Table S1.** Representative categories and types of human RNAs involved in physiological processes and diseases, including cancer

**Table S2.** The implications of defective key components of the DNA damage response mechanisms in the pathogenesis of specific clinical syndromes in humans

## Supporting information


**Supplementary figure legends**

**Full legends for main figures**

**Appendix S1.** Glossary and list of abbreviations

**Figure S1.** The cellular fate following genotoxic insults. The magnitude of the genotoxic insult (low, moderate, excessive) determines cells fate (effective repair, senescence or cell‐death, respectively). Under certain conditions, determined by the stress response parameters (Figure 1B), senescence can present a “dark side”. Likewise, necrosis and/or resistance to apoptosis can build up a pro‐tumorigenic environment (see text for details and references). SASP: senescence‐associated secretory phenotype.

**Figure S2.** Other pathways that contribute to DDR signaling. Accumulating data demonstrate that the DDR function is complemented and/or it cross‐talks with other signaling routes, which also respond to DNA damage 420, 421, 422. To what extent these signaling pathways modulate the DDR function is a subject that has not been fully elucidated. Nevertheless, the implementation of multiple signaling cascades in a DDR network highlights the need for the DNA damage machinery to detect and respond to a wide range of stimuli in various cellular scenarios, underscoring the highly modular organization of the DDR 422. (i) One such signaling pathway involved in DDR is the p38 MAPK. It is one of the three main groups of mitogen‐activated protein kinases (MAPK). It contributes in the G2/M checkpoint, to facilitate DNA repair, via three possible routes: a) the direct phosphorylation of p53, which results in the dissociation of p53 from Mdm2 thus preventing p53 ubiquitination and degradation, b) the association with Gadd45α, which interacts with p53 and increases its stability, and c) the phosphorylation and inhibition of the phosphatase Cdc25B which is responsible for driving the cell cycle through activation of the Cyclin B/Cdc2 complex 53, 423. In addition, p38 MAPK activation can induce G1/S checkpoint in response to a variety of cellular stresses such as osmotic shock or cellular senescence 53, 423. (ii) Hippo signaling pathway is also implicated in the DDR. Further to a wide spectrum of cellular roles, components of the Hippo pathway cooperate with central orchestrators of the DDR, namely the ATR‐Chk1 and ATM‐Chk2 signaling nodes 424, 425. (iii) Wnt/ β catenin pathway, which has important functions in controlling gene expression, cell polarity and adhesion, is also involved in the repair of DNA damage specifically due to oxidative stress, through interaction with DDR at different levels 426, 427, 428. (iv) NOTCH pathway is a highly conserved signaling system that functions in developmental processes related to cell‐fate determination, particularly in stem cells. In mammalian cells, activation of human Notch1 results in reduced ATM signaling in a manner independent of Notch 1 transcriptional activity 429. Notch1 binds directly to the regulatory FATC domain of ATM, thus inhibiting ATM kinase activity 429. (v) An additional paradigm of interaction between DDR and other signaling routes is that of the Hedgehog (Hh) pathway on the DNA repair mechanism. Inhibition of Hh signaling can repress almost all of the DNA repair mechanisms (i.e. BER, NER, MMR and DSB repair including HR and NHEJ) 430. (vi) Immune responses upon DNA damage are supported by a growing body of evidence 24. DNA‐PK, Ku70 and MRE11 are all capable of sensing cytosolic DNA and activating the cGAS‐STING pathway promoting type I and type III interferon‐signaling. Additionally, PARP‐1 and ATM interact with subunits of IκB kinase triggering NF‐κB‐dependent gene expression. ATM and ATR activation is also involved in the upregulation of ligands for the NKG2D receptor upon stalled DNA replication forks. Conversely, key immune system players like the classical cytokine IL‐1α can act as intracellular DNA damage sensors and signal the presence of genotoxic stress 23, 431.

**Figure S3.** Replication‐transcription intermediates and replication fork restart. (A) Replication intermediate lesions harboring single stranded DNA (ssDNA). (i) Uncoupling of the replicative helicase and polymerases results in generation of ssDNA due to excessive unwinding of the template (stalled fork). (L: leading strand; l: lagging strand) (ii) A stalled replication fork may undergo remodeling by creating an intermediate reverse fork also known as “chicken foot” structure: (ii‐1) Direct CtIP processing of the reversed fork may lead to nascent strand ssDNA formation. (ii‐2) Cleavage by SLX4‐docking nucleases generates DNA double strand break that is subsequently followed by resection resulting into nascent strand ssDNA generation. (iii) Unequal branch migration or resection (by CtIP) of a reversed fork can also lead to generation of template ssDNA. (iv) Deregulated firing of clustered origins leads to replication stress and accumulation of gaps in the nascent strands, leaving template ssDNA (see text for details and references) (B) Transcription intermediates. R loops are the predominant transcription generated intermediates and represent a three‐stranded nucleic acid structure that comprises two branches, an RNA–DNA hybrid and an ssDNA. The former can impede completion of replication leading to replication fork stalling, collapse and DSBs formation, while the latter can serve as a substrate to DNA damaging agents and cellular enzymes [APOBEC deaminases (Table 1)] resulting in DNA lesions and/or nicks (see text for details and references). (C) Restart of stalled or collapsed replication forks. Depending on the duration (*how long*) of the replication block, forks can stall or collapse. Restart of stalled forks is promoted by fork remodeling factors, while collapsed forks rely on DSB mediated restart through homologous recombination repair, whereas new origins are concurrently fired (see text for details and references).

**Figure S4.** (A) Nucleolus and rDNA organization. Schematic representation of the rDNA repeats, an organization that renders them susceptible to replication‐transcription collisions (see text for details). PHC: Perinuclear Heterochromatin, FC: Fibrillar Centre, DFC: Dense Fibrillar Component and CC: Granular Component (see text for details and references). (B) Maintaining mitochondrial DNA integrity: Nuclear and mitochondrial DNAs are interdependent. Cartoon of the mitochondrial DNA: D (Displacement)‐loop: a short nucleotide segment complementary to the light (L)‐strand that displaces the heavy (H)‐strand of the mitochondrial DNA. It contains promoters (LSP and HSP) for the RNA transcription from the two strands (heavy and light, respectively) of mitochondrial DNA, possibly involved in the organization of the mitochondrial nucleoid (see text for details and references); LSP: Light strand promoter. The promoter is responsible for gene transcription from the light strand (lower molecular mass) of mitochondrial DNA; HSP: Heavy strand promoter. The promoter is responsible for gene transcription from the heavy strand (higher molecular mass) of mitochondrial DNA. Depending on the magnitude of the mitochondrial DNA damage three levels of repair may take place (see text for details and references).

**Figure S5.** Monitoring Mitosis (DDR surveillance). During M phase checkpoints monitor the proper alignment, segregation and cytokinesis (see lower left panel). In response to a mitotic defect, such as misalignment and/or DSBs, cell fate depends on the context (i.e. p53 status) and the extent of the damage (*how much*): i) low mitotic damage is marked, and repaired in the subsequent cell cycle in the daughter cells (continuous green line corresponds to G1 phase where the majority of DNA lesions are repaired, however, mitotic DNA lesions can also be repaired in S and G2 phase depicted by the dashed green line‐see lower left cell cycle panel), ii) high DNA damage or mitotic spindle defects may lead to mitotic catastrophe or mitotic slippage, which in turn generates aneuploidy and/or CIN. The later can lead to cell death, senescence or development of precancerous lesions. Upon induction of DSBs during mitosis, MRN, and phosphorylated MDC1 and Η2ΑΧ are recruited to the damaged site forming the mitotic DDR foci (see right panel). Notably, 53BP1 and BRCA1 are not recruited to the site of damage blocking NHEJ and HR activation, respectively, preventing telomere fusion (mute DDR). An adverse outcome of mitotic DDR activation is kinetochore‐microtubule stabilization mediated by activation of PLK1 and Aurora kinase A that in turn promotes merotelic attachment and the formation of lagging chromosomes resulting in numerical CIN. However, it is not yet clear under what circumstances activation of mitotic DDR leads to this unfavorable outcome, instead of marking the DNA damage and proceeding to repair in the following cell cycle (marked with a question mark; right lower panel). “P” within red colored circles depicts the two phosphorylation sites of 53BP1 at Threonine‐1609 and Threonine‐1618 that prevent it from recruitment to DDR foci. CIN: chromosomal instability

**Figure S6.** Functional interplay and interdependence of genome and proteome maintenance modules (DDR and PDR surveillance). (A) The PN along with PDR are actively involved in DDR efficiency since by assuring proteome integrity they maintain the functionality of the protein machines that safeguard genome stability. On the other hand, DDR induces a number of proteostatic and/or metabolic adaptations, including suppression of transcription and ribosomal biogenesis, indicating the functional interdependence of the two pathways. These pathways are fully active in young organisms. (B‐C) The age‐related collapse of proteostatic modules functionality and/or expression levels (B) results in the gradual accumulation of non‐functional polypeptides, protein aggregates or lipofuscin, compromising proteome integrity and leading to genomic instability (and thus increased chances for carcinogenesis) as a result of ineffective DNA maintenance and/or repair. Eventually, a vicious cycle may form where a mildly unstable genome accelerates proteome instability due to synthesis of mutated polypeptides that progressively increase the attrition of protein machines resulting in an increasingly stressful cellular landscape that favors the appearance (C) of cellular senescence, cell death or age‐related diseases (e.g. cancer). (D) In normal cells, production of ROS or RNS is neutralized by anti‐oxidant responses while intact PN ensures normal protein turnover. During stress induced premature senescence (Glossary) or in aged tissues the levels of ROS/RNS increase leading to lipid and protein oxidation in the cytoplasm. As this process evolves, oxidized proteins become unfolded and intra‐ and/or inter‐molecular cross links occur, forming non‐degradable oxidized protein aggregates; the latter along with oxidized lipids/lipoproteins, carbohydrate residues and metals form undegradable lipofuscin which accumulates mainly in lysosomes, while only a minor amount is found free in the cytosol. Cytosolic lipofuscin occurs either due to impaired uptake by stalled autophagy or following autophagosome/phagophore rapture. Lipofuscin also inhibits proteasome activity further boosting lipid/lipoprotein oxidation in the cytoplasm.

**Table S1**. Representative categories and types of human RNAs involved in physiological processes and diseases, including cancer

**Table S2.** The implications of defective key components of the DNA damage response mechanisms in the pathogenesis of specific clinical syndromes in humans
