# Supplementary material for: Integrating the DNA damage and protein stress responses during cancer development and treatment
Source: J Pathol. 2018 Jul 19;246(1):12–40. doi: 10.1002/path.5097 (PMC6120562; doi:10.1002/path.5097)
Supplement: Supplementary file 1 — Supplementary figure legends [file PATH-246-12-s008.doc]

**Full legends of main figures**

**Figure 1. (**A) Overview of homeostatic mechanisms: The DNA damage and Protein damage response apparatuses (DDR and PDR) represent the pillars of cellular homeostasis. Their performance under stress conditions determines whether the cell will retain its physiological status, die, senesce or enter a pathophysiological state (see text for details and references). (B) Hallmarks of stressors (see text for details and references). (C) Stress response pathways components. Stress response pathways (SRP) have been evolutionary shaped based on the principle that the cellular *status quo* needs to be maintained. Sensors, signaling cascades (transducers and effectors), regulatory circuits, trafficking modules and repair or destruction sites represent the main SRP components.

**Figure 2. (**A) Synopsis of the types of DNA damage, their estimated frequency and relevant DNA repair mechanisms. (B) The DDR signaling cascade in parallel with other stress response routes (SRPs - see also Figure S2) activates the various checkpoints throughout the cell cycle. A major downstream target of the stress response signaling cascades are the cyclin/CDK complexes. (C) Some, but not all, of the DNA repair mechanisms that function in the nucleus have also been described in the mitochondria (see text for details and references). (*Both short-patch and long-patch BER, **micro-homology recombination).

**Figure 3.** Repair routes for category-S lesions (DDR surveillance). (A) Lesions, such as O^6^-methyl guanine can be subject to direct-protein mediated reversal. (B) Base Excision Repair (BER) and Nucleotide Excision Repair (NER) are high fidelity pathways. BER is responsible for removal of modified bases (*e.g.* oxidized, alkylated or deaminated) and in certain cases deals with SSBs. Initially, DNA glycosylases identify the DNA defect and remove the lesion, generating an abasic site. Next, either the AP-lyase activity of the glycosylases or the apurinic/apyrimidic endonucleases APE1/APE2 incise the DNA strand. Next poly(ADP-Ribose) Polymerase (PARP) is recruited to the abasic site to catalyze the synthesis of a negatively charged poly(ADP-ribose) polymer facilitating chromatin relaxation and access of repair proteins. The resulting SSB can be processed by either short-patch BER (for single nucleotide replacement) or long-patch BER (for 2-10 new nucleotides replacement). PARP is involved mainly in LP-BER. Finally, a polymerase (Pol β/δ/ε) and a ligase (I or III) fill the abasic site and seal the repaired strand, respectively. NER is responsible for repairing helix distorting lesions such as bulky lesions, especially UV induced thymine dimers and 6,4-photoproducts, as well as non-bulky ones. NER is subdivided into Global Genome NER (GG-NER) that recognizes and repairs DNA defects throughout the genome, and Transcription Coupled NER (TC-NER), which identifies DNA lesions that stall DNA transcription. While the two pathways differ in the process of lesion recognition, they share the same route of incision, repair and ligation. DNA damage recognition during GG-NER requires the coordinated action of the XPC-HR23B and XPE-DDB1 complexes, followed by recruitment of the TFIIH complex together with the CAK subcomplex. In TC-NER, this is performed by damaged-stalled RNAPII and the recruitment of the CSB-CSA complexes, followed by the assembly of the core NER components TFIIH, XPA, RPA, XPG and XPF-ERCC1. Repair of nucleotide mis-incorporation is mediated by Mismatch Repair (MMR). Recognition of the mismatched base involves the complexes MutSα (containing MSH2 and MSH6 subunits) and MutSβ (containing MSH2 and MSH3 subunits). Next, incision takes place by the endonuclease activity of the complexes MutLα (containing MLH1 and PMS2 subunits) and MutLγ (containing MLH1 and MLH3 subunits) on the 3’- or 5’- side of the mismatched base on the discontinuous strand, while the role of MutLβ (containing MLH1 and PMS1 subunits) is less well understood. Then, EXO I excises the resulting DNA segment, in cooperation with the single-stranded DNA-binding protein RPA. Finally, polymerase and ligase activities sequentially fill and seal the affected strand. (C) Translesion Synthesis repair (TLS) pathway. Following DNA damage, the RAD 6/18 complex catalyzes PCNA mono-ubiquitination. Next, normal high-fidelity DNA replication polymerases are substituted by TLS polymerases that bypass the lesion through inserting a nucleotide opposite it. Finally, TLS polymerases are substituted by high-fidelity ones to continue the synthesis process.

**Figure 4.** Repairing category-D lesions (DDR surveillance). Double strand breaks (DSBs) and interstrand crosslinks (ICLs) represent category-D defects. DSBs initiate a sophisticated DNA damage response and repair (DDR/R) cascade (see text for details and references). Briefly, sensors and mediators are recruited along with chromatin remodeling factors on the flanking sites of the lesion, generating foci that “tag” the DSB presence. A key event for foci establishment is ATM-mediated phosphorylation of the histone variant H2AX (γH2AX). Next, the signal is amplified (signal spreading) followed by a coordinated systemic and local effect, mediated by transducers and effectors. At the systemic level the DDR/R determines cell fate through various cycle checkpoints (see text for details and references as well as Figure 3). At the local level, DSB lesions are repaired by non-homologous end joining (NHEJ) or homologous recombination repair (HRR) (left panel). NHEJ relies on short homologous DNA sequences (microhomologies) present at overhangs on the ends of DSBs to guide repair. During NHEJ, promoted by the signaling mediator 53BP1, DNA end breaks are recognized and bound by the Ku70/80 heterodimer. Subsequently, DNA-PK binds the two ends and along with ARTEMIS performs end resection. The process is terminated by synthesis and ligation. HRR occurs only in S and G2 phases when the sister chromatid is available. Briefly, DSBs are recognized by the MRN complex (MRE11/RAD59/NBS), the ends are recessed by CtIP and EXO I and the resulting single-stranded 3' overhangs are coated with RPA, facilitating Rad51 loading. Subsequently, the Rad51 single-stranded DNA nucleoprotein filaments invade the homologous chromatid, forming a D-loop. The invading 3’ strand extends along the recipient homologous DNA duplex by DNA polymerase (Pol δ). At this point, HRR can follow two distinct routes. One involves the double-strand break repair (DSBR) pathway, during which after strand invasion and synthesis, the other DSB end can be bound leading to the generation of a two Holliday junctions (HJs) intermediate (see area with red dashed border). Following gap DNA synthesis and ligation, the intermediate structure is resolved at the HJs in a non- or crossover (exchange between sister chromatids) manner. The other pathway is synthesis-dependent strand annealing (SDSA), during which after strand invasion and synthesis, the extended single-strand end is annealed to the ssDNA on the second complementary side of the DSB, followed by DNA gap-filling and ligation (see area with red dashed border). The repair product from SDSA is always non-crossover. Both DSBR and SDSA are generally considered error-free mechanisms. Alternative HRR modules that involve recombination across regions with incomplete homology can also take place and are error prone repair processes. The break-induced repair (BIR) pathway is a Rad52-dependent homology repair route of one sided DSBs. While the initial steps are similar to DSBR and SDSA, up to formation of the D-loop, this is not dissolved unless the whole missing chromosomal arm is replicated. The single-strand annealing (SSA) pathway requires only a single DNA duplex, using repeat sequences flanking the DSB as the homologous recombination sequences needed for repair. DNA damage recognition (MRN complex) and recession (CtIP and EXO I) generates single-stranded 3' overhangs that are coated with the RPA protein to prevent overhangs from sticking between them. Next, Rad52 binds the repeat sequences on each side of the break and aligns them to enable their annealing. Subsequently, the 3' non-homologous flaps are excised by XPF/ERCC1 and finally gaps are filled and ligated. Microhomology-mediated end joining (MMEJ), or alternative nonhomologous end-joining (Alt-NHEJ), is a repair variant that relies on the use of 5–25 base pair (bp) microhomologous sequences to align DSB ends before joining, thereby resulting in deletions flanking the break. It does not require ATM activation, instead it is initiated by a limited resection of DNA DSB ends by the MRN complex and CtIP. PARP1 or PARP2 homo- or heterodimers are recruited at resected DNA DSB ends, together with DNA polymerase theta and FEN1 5'-flap endonuclease. Finally, DNA ligase 3 (LIG 3)/XRCC1 seals the strand. Interstrand crosslinks (ICLs) are recognized and resolved by the Fanconi Anemia (FA) pathway, a highly coordinated repair process that involves the coordinated action of the FA modules with the sequential action of TLS, NER and HRR components to resolve ICLs (see text for details and references).

**Figure 5.** A model depicting how oncogene induced replication stress aids the progressive formation of certain hallmarks of cancer (early events: steps 1-5), while paving the way for, angiogenesis, evasion from immune surveillance, invasion and metastasis (late events-6). Specifically, oncogenic activation acts as a force that pushes the cell away from its equilibrium point. Activated oncogenes lead to replication stress either directly by deregulating the replication machinery or indirectly via affecting metabolic pathways (1, 2). DNA lesions resulting from oncogene activation stimulate the DNA damage response pathway to promote repair and impose the tumorigenic barriers of apoptosis and senescence. In the event of a perturbed DNA damage response, cells accumulate genomic instability, proteotoxic and mitotic stress (3, 4). Failure to elicit apoptosis or escape from senescence (5) can lead to oncogenic transformation and primary tumor formation. These early events can also pave the way for later events including angiogenesis, evasion from immune surveillance, invasion and metastasis. This is a link that requires further investigation (see text for details). Strike, hourglass: over time, genomic instability shapes the stages for cancer progression. ?: a potential link that requires further investigation (see text for details and references).

**Supplementary figure legends**

**Figure S1.** The cellular fate following genotoxic insults. The magnitude of the genotoxic insult (low, moderate, excessive) determines cells fate (effective repair, senescence or cell-death, respectively). Under certain conditions, determined by the stress response parameters (Figure 1B), senescence can present a “dark side”. Likewise, necrosis and/or resistance to apoptosis can build up a pro-tumorigenic environment (see text for details and references). SASP: senescence-associated secretory phenotype.

**Figure S2.** Other pathways that contribute to DDR signaling. Accumulating data demonstrate that the DDR function is complemented and/or it cross-talks with other signaling routes, which also respond to DNA damage [420-422] (reference numbers refer to the main text list). To what extent these signaling pathways modulate the DDR function is a subject that has not been fully elucidated. Nevertheless, the implementation of multiple signaling cascades in a DDR network highlights the need for the DNA damage machinery to detect and respond to a wide range of stimuli in various cellular scenarios, underscoring the highly modular organization of the DDR [422]. (i) One such signaling pathway involved in DDR is the p38 MAPK. It is one of the three main groups of mitogen-activated protein kinases (MAPK). It contributes in the G2/M checkpoint, to facilitate DNA repair, via three possible routes: a) the direct phosphorylation of p53, which results in the dissociation of p53 from Mdm2 thus preventing p53 ubiquitination and degradation, b) the association with Gadd45α, which interacts with p53 and increases its stability, and c) the phosphorylation and inhibition of the phosphatase Cdc25B which is responsible for driving the cell cycle through activation of the Cyclin B/Cdc2 complex [53,423]. In addition, p38 MAPK activation can induce G1/S checkpoint in response to a variety of cellular stresses such as osmotic shock or cellular senescence [53,423]. (ii) Hippo signaling pathway is also implicated in the DDR. Further to a wide spectrum of cellular roles, components of the Hippo pathway cooperate with central orchestrators of the DDR, namely the ATR-Chk1 and ATM-Chk2 signaling nodes [424,425]. (iii) WNT / β catenin pathway, which has important functions in controlling gene expression, cell polarity and adhesion, is also involved in the repair of DNA damage specifically due to oxidative stress, through interaction with DDR at different levels [426-428]. (iv) Notch pathway is a highly conserved signaling system that functions in developmental processes related to cell-fate determination, particularly in stem cells. In mammalian cells, activation of human Notch1 results in reduced ATM signaling in a manner independent of Notch 1 transcriptional activity [429]. Notch1 binds directly to the regulatory FATC domain of ATM, thus inhibiting ATM kinase activity [429]. (v) An additional paradigm of interaction between DDR and other signaling routes is that of the Hedgehog (Hh) pathway on the DNA repair mechanism. Inhibition of Hh signaling can repress almost all of the DNA repair mechanisms (i.e. BER, NER, MMR and DSB repair including HR and NHEJ) [430]. (vi) Immune responses upon DNA damage are supported by a growing body of evidence [24]. DNA-PK, Ku70 and MRE11 are all capable of sensing cytosolic DNA and activating the cGAS-STING pathway promoting type I and type III interferon-signaling. Additionally, PARP-1 and ATM interact with subunits of IκB kinase triggering NF-κB-dependent gene expression. ATM and ATR activation is also involved in the upregulation of ligands for the NKG2D receptor upon stalled DNA replication forks. Conversely, key immune system players like the classical cytokine IL-1α can act as intracellular DNA damage sensors and signal the presence of genotoxic stress [23, 431].

**Figure S3.** Replication-transcription intermediates and replication fork restart. (A) Replication intermediate lesions harboring single stranded DNA (ssDNA). (i) Uncoupling of the replicative helicase and polymerases results in generation of ssDNA due to excessive unwinding of the template (stalled fork). (L: leading strand; l: lagging strand) (ii) A stalled replication fork may undergo remodeling by creating an intermediate reverse fork also known as “chicken foot” structure: (ii-1) Direct CtIP processing of the reversed fork may lead to nascent strand ssDNA formation. (ii-2) Cleavage by SLX4-docking nucleases generates DNA double strand break that is subsequently followed by resection resulting into nascent strand ssDNA generation. (iii) Unequal branch migration or resection (by CtIP) of a reversed fork can also lead to generation of template ssDNA. (iv) Deregulated firing of clustered origins leads to replication stress and accumulation of gaps in the nascent strands, leaving template ssDNA (see text for details and references) (B) Transcription intermediates. R loops are the predominant transcription generated intermediates and represent a three-stranded nucleic acid structure that comprises two branches, an RNA-DNA hybrid and an ssDNA. The former can impede completion of replication leading to replication fork stalling, collapse and DSBs formation, while the latter can serve as a substrate to DNA damaging agents and cellular enzymes [APOBEC deaminases (Table 1)] resulting in DNA lesions and/or nicks (see text for details and references). (C) Restart of stalled or collapsed replication forks. Depending on the duration (*how long*) of the replication block, forks can stall or collapse. Restart of stalled forks is promoted by fork remodeling factors, while collapsed forks rely on DSB mediated restart through homologous recombination repair, whereas new origins are concurrently fired (see text for details and references).

**Figure S4.** (A) Nucleolus and rDNA organization. Schematic representation of the rDNA repeats, an organization that renders them susceptible to replication-transcription collisions (see text for details). PHC: Perinuclear Heterochromatin, FC: Fibrillar Centre, DFC: Dense Fibrillar Component and CC: Granular Component (see text for details and references). (B) Maintaining mitochondrial DNA integrity: Nuclear and mitochondrial DNAs are interdependent. Cartoon of the mitochondrial DNA: D (Displacement)-loop: a short nucleotide segment complementary to the light (L)-strand that displaces the heavy (H)-strand of the mitochondrial DNA. It contains promoters (LSP and HSP) for the RNA transcription from the two strands (heavy and light, respectively) of mitochondrial DNA, possibly involved in the organization of the mitochondrial nucleoid (see text for details and references); LSP: Light strand promoter. The promoter is responsible for gene transcription from the light strand (lower molecular mass) of mitochondrial DNA; HSP: Heavy strand promoter. The promoter is responsible for gene transcription from the heavy strand (higher molecular mass) of mitochondrial DNA. Depending on the magnitude of the mitochondrial DNA damage three levels of repair may take place (see text for details and references).

**Figure S5.** Monitoring Mitosis (DDR surveillance). During M phase checkpoints monitor the proper alignment, segregation and cytokinesis (see lower left panel). In response to a mitotic defect, such as misalignment and/or DSBs, cell fate depends on the context (i.e. p53 status) and the extent of the damage (*how much*): i) low mitotic damage is marked, and repaired in the subsequent cell cycle in the daughter cells (continuous green line corresponds to G1 phase where the majority of DNA lesions are repaired, however, mitotic DNA lesions can also be repaired in S and G2 phase depicted by the dashed green line-see lower left cell cycle panel), ii) high DNA damage or mitotic spindle defects may lead to mitotic catastrophe or mitotic slippage, which in turn generates aneuploidy and/or CIN. The later can lead to cell death, senescence or development of precancerous lesions. Upon induction of DSBs during mitosis, MRN, and phosphorylated MDC1 and Η2ΑΧ are recruited to the damaged site forming the mitotic DDR foci (see right panel). Notably, 53BP1 and BRCA1 are not recruited to the site of damage blocking NHEJ and HR activation, respectively, preventing telomere fusion (mute DDR). An adverse outcome of mitotic DDR activation is kinetochore-microtubule stabilization mediated by activation of PLK1 and Aurora kinase A that in turn promotes merotelic attachment and the formation of lagging chromosomes resulting in numerical CIN. However, it is not yet clear under what circumstances activation of mitotic DDR leads to this unfavorable outcome, instead of marking the DNA damage and proceeding to repair in the following cell cycle (marked with a question mark; right lower panel). “P” within red colored circles depicts the two phosphorylation sites of 53BP1 at Threonine-1609 and Threonine-1618 that prevent it from recruitment to DDR foci. CIN: chromosomal instability

**Figure S6.** Functional interplay and interdependence of genome and proteome maintenance modules (DDR and PDR surveillance). (A) The PN along with PDR are actively involved in DDR efficiency since by assuring proteome integrity they maintain the functionality of the protein machines that safeguard genome stability. On the other hand, DDR induces a number of proteostatic and/or metabolic adaptations, including suppression of transcription and ribosomal biogenesis, indicating the functional interdependence of the two pathways. These pathways are fully active in young organisms. (B-C) The age-related collapse of proteostatic modules functionality and/or expression levels (B) results in the gradual accumulation of non-functional polypeptides, protein aggregates or lipofuscin, compromising proteome integrity and leading to genomic instability (and thus increased chances for carcinogenesis) as a result of ineffective DNA maintenance and/or repair. Eventually, a vicious cycle may form where a mildly unstable genome accelerates proteome instability due to synthesis of mutated polypeptides that progressively increase the attrition of protein machines resulting in an increasingly stressful cellular landscape that favors the appearance (C) of cellular senescence, cell death or age-related diseases (e.g. cancer). (D) In normal cells, production of ROS or RNS is neutralized by anti-oxidant responses while intact PN ensures normal protein turnover. During stress induced premature senescence (see Glossary) or in aged tissues the levels of ROS/RNS increase leading to lipid and protein oxidation in the cytoplasm. As this process evolves, oxidized proteins become unfolded and intra- and/or inter-molecular cross links occur, forming non-degradable oxidized protein aggregates; the latter along with oxidized lipids/lipoproteins, carbohydrate residues and metals form undegradable lipofuscin which accumulates mainly in lysosomes, while only a minor amount is found free in the cytosol. Cytosolic lipofuscin occurs either due to impaired uptake by stalled autophagy or following autophagosome/phagophore rapture. Lipofuscin also inhibits proteasome activity further boosting lipid/lipoprotein oxidation in the cytoplasm.
