# Supplementary material for: Integrating the DNA damage and protein stress responses during cancer development and treatment
Source: J Pathol. 2018 Jul 19;246(1):12–40. doi: 10.1002/path.5097 (PMC6120562; doi:10.1002/path.5097)
Supplement: Supplementary file 2 — Full legends for main figures [file PATH-246-12-s005.docx]

**Note added in proof:**

While this review was in proof stage three important studies were published relevant to the context of this manuscript and we would like briefly to present them in this note.

Novel mechanistic details on how perturbed replication results in genomic instability came out from Halazonetis and Bartek labs. Morgane and Halazonetis in a genome wide study mapped replication and transcription before and after oncogene activation [[1](#_ENREF_1)]. They showed that under physiologic conditions transcription suppresses intragenic origin firing. However, upon oncogene induction premature entry in S phase allows intragenic origin firing within transcribed genes. These origins are prone to collapse in DSBs forming hotspots for chromosomal rearrangements and contributing in genomic instability. This model of transcription acting in supressing intragenic origin firing within transcribed genes offers a new understanding on how shortening of G1 phase upon oncogene activation promotes genomic instability.

Maya-Mendoza *et al*. showed that not only discrepancies in initiation of DNA replication but also aberrant elongation results in replication stress by finding that there is a threshold of fork speed beyond which cells trigger DDR [[2](#_ENREF_2)]. This study revealed an unexpected role of PARP1 as a sensor of replication stress at ongoing forks in targeting p53, promoting accumulation of p21^WAF1/Cip1^ expression and therefore eliminating progression of defective forks. Treatment with the PARP inhibitor olaparib resulted in increased fork speed, challenging the exiting model that PARP inhibition results in fork collapse due to prolong stalling and offering a new mechanistic insight on how PARP inhibitors function in synthetic lethality approaches.

We recently showed that location of lesions in the genome is important for the impact of DNA damage to genomic stability [[3](#_ENREF_3)]. We showed that breaks in the rDNA result in changes in local chromatin configuration with the establishment of H2B phosphorylation at Serine 14 via the MST2 kinase promoting inhibition of Pol I transcriptional activity. This study adds mechanistic details on the recently emerging field of the nucleolar responses to DNA damage and on how the DNA damage response is organized in this hotspot of genomic instability.

Related references:

1. Macheret M, Halazonetis T D. Intragenic origins due to short G1 phases underlie oncogene-induced DNA replication stress. *Nature* 2018; **555**: 112-116.

2. Maya-Mendoza A, Moudry P, Merchut-Maya JM, *et al*. High speed of fork progression induces DNA replication stress and genomic instability. *Nature* 2018; [Epub ahead of print].

3. Pefani DE, Tognoli ML, Pirincci Ercan D, *et al*. MST2 kinase suppresses rDNA transcription in response to DNA damage by phosphorylating nucleolar histone H2B. *The EMBO J* 2018; **pii:** e98760.
