# Supplementary material for: Integrating the DNA damage and protein stress responses during cancer development and treatment
Source: J Pathol. 2018 Jul 19;246(1):12–40. doi: 10.1002/path.5097 (PMC6120562; doi:10.1002/path.5097)
Supplement: Supplementary file 3 — Appendix S1. Glossary and list of abbreviations [file PATH-246-12-s011.docx]

**Glossary**

*Note: all terms are highlighted by an asterisk when appearing for the first time in the main text

**26S proteasome:** a large multi-catalytic ATP-dependent protease complex involved in the regulated degradation of ubiquitinated proteins in the cell

**AMPK (AMP-activated protein kinase):** a highly conserved heterotrimer that senses intracellular adenosine nucleotide levels, regulating cellular energy metabolism

**Anaphase promoting complex (APC/C):** also called cyclosome, is a large multi-protein complex with E3 ubiquitin ligase activity that targets mitotic cyclin B and securin for degradation, triggering thus sister chromatid separation

**Aneuploidy:** a state of gain or loss of whole chromosomes is called whole aneuploidy. Partial excess or deficiency of chromosomes refers to segmental aneuploidy. It should not be confused with polyploidy that refers to the presence of multiple complete sets of chromosomes and to CIN which corresponds to rate of chromosomal changes

**APOBEC (apolipoprotein B mRNA editing enzyme, catalytic subunit):** a family of cytidine deaminases, inducing the conversion of cytosine to uracil at single strand nucleic acids. They are involved in RNA editing and function as viral protecting agents. By deaminating cytosine in ssDNA they act as a source of DNA damage causing hypermutations at cancer genome

**Aurora-A:** a protein kinase that associates with the centrosome and the spindle microtubules during mitosis and plays a critical role in various mitotic events including establishment of the mitotic spindle, centrosome duplication, centrosome separation as well as maturation, chromosomal alignment, spindle assembly checkpoint and cytokinesis

**Aurora-B:** a protein kinase that is essential for the correct attachment of the mitotic spindle to the kinetochore. It is a key component of abscission/cytokinesis checkpoint

**Autophagy:** a highly conserved catabolic cellular process by which damaged intracellular proteins and organelles -the cargo- are degraded in the lysosomes and are recycled to meet the metabolic needs of the cell. There are three types of autophagy in eukaryotic cells, depending on the mechanism of delivery of the damaged entities into the lysosomes: microautophagy, chaperone-mediated and macroautophagy. The latter is a multistep process characterized by the biogenesis of double-membrane vesicles, the autophagosomes, through the initiation, nucleation and elongation stages. The mature autophagosomes carrying the cargo fuse with lysosomes for degradation of the cargo. Among the key regulatory factors of autophagy are Beclin-1 during nucleation, the microtubule-associated protein 1 light chain 3 (LC3) in the elongation step and sequestosome 1/p62 (SQSTMQ/p62), the best characterized autophagy substrate identifying and delivering ubiquitinated cargo into the lysosomes

**Autophagy related proteins (Atg):** highly conserved proteins involved in autophagosome formation that starts from the assembly of the autophagy initiation complex and terminates with autophagosome membrane elongation

**BASC complex (BRCA1-associated genome surveillance complex):** A multi-protein complex that is assembled on double strand breaks and includes BRCA1, ATM, the BLM helicase, the RAD50-MRE11-NBS1 complex, MSH2, MSH6 and MLH1

**BLM (Bloom syndrome protein):** protein related to the RecQ subset of DExH box-containing DNA helicases and has both DNA-stimulated ATPase and ATP-dependent DNA helicase activities; it suppresses sister chromatid exchange, while *BLM* mutations cause Bloom syndrome. Bloom syndrome is characterized by growth retardation, microcephaly and increased risk of cancer

**Caretaker genes:** type of tumor suppressor genes, essential for protecting the integrity of the genome (including genes encoding for proteins involved in DNA repair and surveillance of mitosis). Mutations in caretaker genes promote genomic instability

**CDC20 (Cell Division Cycle 20):** an essential regulator of cell division activating APC/C which initiates chromatid separation and entrance into anaphase

**Centrosome:** is a major microtubule-organizing centre of the cell, consisting of two centrioles surrounded by pericentriolar material; the centrosome is duplicated during S-phase and in mitosis the two centrosomes form the poles of the mitotic spindle, which promotes the proper segregation of the chromosomes into two daughter cells

**Chaperones:** are responsible for proper polypeptide folding, unfolding and remodeling, as well as for the assembly of the complicated protein machines and/or the delivery of unfolded polypeptides to degradation machineries. Three types of chaperones are **foldases, holdases** and **disaggregases**. Foldases are a class of ATP-dependent molecular chaperones that assist non-covalent folding of proteins. Holdases are a class of ATP-independent molecular chaperones which can recognize and stabilize partially folded proteins, preventing their aggregation and presenting client proteins to foldases. Disaggregases are ATP-dependent molecular chaperones that extract polypeptides from aggregates

**Checkpoint activation:** surveillance mechanism triggering transient or sustained cell cycle blockade upon DNA damage to ensure DNA repair before proceeding to the next phase of the cell cycle. Alternatively, upon non-repairable damage, it promotes cell death

**Chromatin modifications and remodeling:** highly dynamic modification of chromatin architecture, employing remodelers that move, eject or restructure nucleosomes utilizing the energy provided by ATP hydrolysis. They regulate gene expression, DNA replication and repair

**Chromosomal instability (CIN):** is an elevated rate of chromosomal imbalances. It can be classified as structural CIN, referring to the ongoing rate of amplifications, deletions or translocations; and numerical CIN related with a high rate of gain or losses of chromosomes

**Chromothripsis:** a phenomenon characterized by extensive genomic rearrangements that occur in a single catastrophic event restricted to one or a few chromosomes

**Claspin:** an essential upstream regulator of checkpoint kinase 1 (Chk1), triggering a checkpoint arrest of the cell cycle in response to replicative stress or DNA damage. Also required for efficient DNA replication during a normal S phase

**Clastogens:** mutagenic agents inducing disruption or breakages of chromosomes, leading to deletions, gains or rearrangements

**Clustered DNA lesions:** two or more bistranded DNA lesions occurring within 1-10 base pairs, including double strand breaks (DSB) with associated base lesions or abasic (AP) sites, and non-DSB clusters comprised of base lesions, AP sites and single strand breaks

**Cohesin ring:** a multisubunit protein complex that keeps sister chromatids together

**Common fragile sites (CFS):** late replicating areas in the genome that are susceptible to double strand breaks upon replication stress. They are targeted early during carcinogenesis

**CtIP:** endonuclease that cooperates with the MRE11-RAD50-NBN (MRN) complex in DNA-end resection, the first step of double-strand break (DSB) repair through the homologous recombination (HR) pathway, playing a key role in activation of CHK1 kinase to induce the cell cycle checkpoint

**Cyclin-B (CCNB1):** a member of the cyclin family of proteins involved in mitosis. The gene product complexes with CDK1 (cdc2) for proper control of the G2/M transition phase of the cell cycle. Degradation of Cyclin-B by APC/C is necessary for mitotic exit and completion of the cell cycle

**Cyclin/CDK complex:** protein complex formed by the association of an inactive catalytic subunit of a CDK with a regulatory subunit, *i.e*. a cyclin; the formed complex is in an activated state

**Cyclin-dependent kinases (CDKs):** a family of serine/threonine kinases whose activity depends on non-catalytic regulatory subunits termed cyclins. They regulate cell cycle progression.

**Cytokinesis (**or **Abscission) Checkpoint:** quality control mechanism monitoring cytokinesis. It arrests abscission due to the presence of chromatin in the intercellular bridge. Besides high membrane tension, DNA replication stress and defective assembly of nuclear pore induce arrest of cytokinetic cells. It relies on prolonged Aurora-B

**DNA2** **nuclease:** enzyme involved in DNA replication and repair, by processing DNA double-strand breaks, Okazaki fragments and stalled replication forks; it requires ssDNA ends and is dependent on the ssDNA-binding protein RPA

**D-loop:** a term used in two different contexts. i) found in mitochondrial DNA as a replication intermediate, ii) a recombination intermediate during homologous recombination

**Downstream transducer:** proteins belonging to the DDR/R machinery that are recruited by upstream transducers and amplify the signaling though activation of effectors

**Effectors:** proteins belonging to the DDR/R machinery activated by transducers, regulating cell cycle progression and arrest, DNA repair systems, cellular senescence and apoptosis

**eNoSC (energy-dependent Nucleolar Silencing Complex):** chromatin remodeling complex encompassing the NAD+ dependent deacetylase SIRT1, the methyltransferase SUV39H1 and the NP protein nucleomethylin, which regulates rDNA transcription according to NAD+/NADH ratio

**Epithelial-mesenchymal transition (EMT):** a developmental process that allows an epithelial cell to undergo multiple molecular-biochemical changes, enabling a mesenchymal cell phenotype. Ill-timed induction of EMT is associated with pathophysiological conditions

**EXO1:** 5'->3' double-stranded DNA exonuclease, which may also possess a cryptic 3'->5' double-stranded DNA exonuclease activity. Functions in DNA mismatch repair (MMR) to excise mismatch-containing DNA tracts directed by strand breaks located either 5' or 3' to the mismatch. Also exhibits endonuclease activity against 5'-overhanging flap structures similar to those generated by displacement synthesis when DNA polymerase encounters the 5'-end of a downstream Okazaki fragment. Required for somatic hypermutation (SHM) and class switch recombination (CSR) of immunoglobulin genes. Essential for male and female meiosis

**FA (Fanconi Anemia) Core complex:** a multi-subunit E3 ubiquitin ligase, recruited upon interstrand crosslink DNA lesions. It activates the FA pathway by promoting mono-ubiquitination of FANCD2 and FANCI

**Fanconi Anemia:** Rare autosomal recessive disease characterized by developmental abnormalities, aplastic anemia and cancer susceptibility

**Fission (mitochondrial):** separation of mitochondrial membranes and rejoining allowing the partitioning of mitochondrial content

**Fusion (mitochondrial):** fusion of outer mitochondrial membranes allowing efficient mixing of mitochondrial content

**GI (Genomic instability):** A hallmark of cancer. GI refers to high rate of structure and number variations; also called structural and numerical chromosome instability (CIN), respectively. Microsatellite instability (MSI, also known as MIN) is another form of GI characterized by alterations in the number of oligonucleotide repeats present within microsatellite sequences

**G3BP1:** Ras GTPase-activating protein-binding protein 1, which is an effector of stress granule assembly. It has also a phosphorylation-dependent sequence-specific endoribonuclease activity *in vitro*. It preferentially unwinds partial DNA and RNA duplexes

**G-quadruplex DNA:** higher-order DNA and RNA structures formed from G-rich sequences that are built around tetrads of hydrogen-bonded guanine bases

**Heterochromatin-associated proteins:** non-histone proteins tightly associated with heterochromatic regions promoting gene silencing and transcriptional regulation

**HLTF:** a double-stranded DNA translocase, member of the SWI/SNF family, possessing intrinsic ATP-dependent nucleosome-remodeling activity; it plays a role in error-free post-replication repair of damaged DNA and maintains genomic stability through acting as a ubiquitin ligase for 'Lys-63'-linked polyubiquitination of chromatin-bound PCNA

**Hypomorphic:** describes a mutation that causes a partial loss of gene function

**Inner nuclear membrane:** membrane that separates the nuclear matrix from the intermembrane space; in mammals, it is associated with heterochromatin and the nuclear lamina providing structural support of the nucleus, regulating chromatin organization and gene expression

**Kinetochore:** A protein complex located at the centromere of each chromosome, essential for chromosome attachment to the mitotic spindle

**Lagging chromosomes:** chromosomes lagging behind at the spindle equator while all the other chromosomes move toward the spindle poles, leading to numerical CIN

**Mediators:** the molecular link between sensors and other sensors and/or transducers

**Merotelic attachments:** a mitotic error in which a single kinetochore is attached to microtubules emanating from both spindle poles; they can persist into anaphase and induce chromosome missegregation in the form of lagging chromosomes

**Micronuclei:** Aberrant extra-nuclear structures that contain fragmented chromosomal material and characterize structural chromosomal instability

**Mitophagy:** removal of damaged mitochondria through mitochondria-specific autophagy

**Mitostasis:** a homeostatic signaling network establishing the maintenance of an appropriately distributed pool of healthy mitochondria

**Mitotic catastrophe:** a type of cell death occurring during mitosis as a result of increased DNA damage or deranged spindle formation coupled with inactivation of various checkpoints that would normally arrest progression into mitosis

**Mitotic slippage:** term referred to the mitotic arrest evasion and proceeding to interphase

**MLL4:** an essential subunit of the histone H3 Lys4 (H3K4)-methylation complexes, activating transcription

**MRE11:** Nuclease subunit of the MRN complex, possessing endonuclease and 3’-5’ exonuclease activity. It binds single and double-stranded telomeric DNA and G-quadruplex DNA. Involved in multiple processes during meiosis and DNA repair including reciprocal recombination, DNA double-strand break processing and repair via nonhomologous end joining or break-induced replication, base-excision repair and regulation of transcription

**Mutational signature:** a characteristic mutational pattern in the cancer genome that reflects the error-prone repair process that took place

**NoRC (Nucleolar Remodeling Complex):** chromatin remodeling complex containing the ATPase SNF2h (sucrose non-fermenting protein complex 2) and a large subunit named TIP5 (TTF-I-interacting protein 5, also known as BAZ2A), which silences a fraction of rDNA by establishing a heterochromatic structure at the rDNA promoter

**Nucleolar Organizing regions (NORs):** chromosomal landmarks that consist of tandem repeated sequences of ribosomal genes (rRNA). In eukaryotes, each unit consists of three genes coding for 18S, 5.8S and 28S ribosomal RNA. These genes are separated by two intergenic spacers and an external transcribed spacer. Head-to-tail repeats of these units form distinct clusters on one to several chromosome pairs

**Nucleosome remodeler:** ATP-dependent chromatin remodeling factor altering the positions of nucleosomes along DNA and generating superhelical torsion, rendering DNA either accessible or inaccessible to interacting proteins

**NuRD (Nucleosome Remodeling and Deacetylation):** chromatin remodeling complex that consists of various protein subunits with ATP-dependent remodeling and histone deacetylase activity

**Oncogenes:** group of genes capable of inducing cell transformation; there are mutant forms of proto-oncogenes

**PGC1-a (Peroxisome proliferator-activated receptor gamma coactivator 1-alpha):** a transcriptional coactivator which is a central regulator of mitochondrial biogenesis and function

**Polo-like kinase 1 (PLK1):** a serine/threonine kinase with pleiotropic functions acting as a pivotal regulator of cell division

**Post-translational modifications (PTM):** a term referred to the covalent and generally enzymatic modification of proteins introduced after translation from RNA, such as phosphorylation, ubiquitinylation, sumoylation, methylation, acetylation, glycosylation, poly(ADP-ribosyl)ation

**PTIP:** multifunctional DNA repair factor that interacts with phosphorylated Ser25 of 53BP1 through its tandem BRCT domains, regulating ATM association. PTIP is also part of the MLL3/MLL4 histone H3 Lys4 methyltransferase complexes, involved in transcriptional regulation

**Quantum Bioinformatics:** a mathematic formalism used in quantum information theory, applied to model behavioral of biological systems

**RAD52:** The protein encoded by this gene is important for DNA double-strand break repair and homologous recombination. Binds single-stranded DNA ends and mediates the DNA-DNA interaction necessary for the annealing of complementary DNA strands. It was also found to interact with DNA recombination protein RAD51, which suggested its role in RAD51 related DNA recombination and repair

**RECQ-1:** a member of the RecQ DNA helicase family involved in various types of DNA repair, including mismatch repair, nucleotide excision repair and direct repair; additionally, it is involved in the replication fork restarting, processing of Holliday junctions, suppression of sister chromatid exchanges and telomere maintenance

**Repetitive DNA sequences:** patterns of non-coding DNA sequences that occur in multiple copies throughout the genome

**Replication fork barrier (RBF):** an ∼100-bp DNA sequence located near the 3′ end of the rRNA gene repeats, inhibiting replication forks in the direction opposite to rDNA transcription

**Replication fork stalling, collapse:** a term that refers to the slowing (stalling) and, if not repaired, to the dissociation of the replisome from the DNA (collapse)

**Replication licensing:** a regulatory system ensuring that DNA replication takes place once per cell cycle. Key components are the ORC complex as well as the Cdt1 and Cdc6 replication licensing factors

**Replication stress:** any event that impedes normal DNA synthesis

**Replication timing (RT):** a term referred to the temporal order of genomic segments’ replication

**Ribosomal stress:** phenomenon characterized by impairment of nucleolar structure or disruption of ribosome biogenesis

**RNA binding proteins (RBPs):** proteins that bind to the double or single stranded RNA in cells, forming ribonucleoprotein complexes. They participate in post-transcriptional processes in all eukaryotes, such as splicing regulation, mRNA transport and modulation of mRNA translation and decay

**R-loop:** a three-stranded nucleic acid structure composed of a DNA-RNA hybrid and the associated non-template single-stranded DNA

**RNA-polymerase I (RNAPI):** eukaryotic RNA polymerase responsible for rRNA production

**RNA-polymerase II (RNAPII):** eukaryotic RNA polymerase responsible for mRNA and most snRNA and microRNA production

**SAC (Spindle Assembly Checkpoint):** quality control mechanism that prevents anaphase until all chromosomes are properly attached to the spindle

**SAC machinery:** protein complex responsible for SAC signaling, including MAD1 (mitotic arrest deficient 1), MAD2, MPS1 (monopolar spindle 1), BUB1 (budding uninhibited by benzimidazole 1), BUB3, BUBR1, the ZW10-ZWINT-ZWILCH complex and CENPE (centromere protein-E), Aurora B and C (Ipl1)

**Securin (PTTG1):** a chaperone that inhibits separase

**Senescence Associated Secretory Phenotype (SASP):** also known as the senescence-messaging secretome (SMS) is a complex pro-inflammatory response of the senescent cells. It is mediated by the transcription factors nuclear factor‑κB (NF‑κB) and CCAAT/ enhancer binding protein-β (CEBPβ), and includes a wide range of factors, such as the secretion of pro-inflammatory cytokines (interleukin‑6 (IL‑6) and IL‑8), chemokines (monocyte chemoattractant proteins (MCPs) and macrophage inflammatory proteins (MIPs)), growth factors (transforming growth factor-β (TGFβ) and granulocyte–macrophage colony-stimulating factor (GM‑CSF)) and proteases

**Senescence (cellular):** a stress response mechanism characterized by irreversible cell cycle arrest, apoptosis resistance, metabolic activity and a senescence-associated secretory phenotype (SASP). It is a fundamental biological process involved in normal embryonic and adult life and implicated in various age-related disorders such as cancer and aging

**Sensors:** proteins belonging to the DDR/R machinery capable of binding and recognizing DNA structural alterations and signaling different types of repair DNA damage

**Separase:** a cysteine protease that triggers anaphase by cleaving the cohesion rings

**Shelterin:** a six-subunit protein complex that associates specifically with mammalian telomeres and allows cells to distinguish the natural ends of chromosomes from sites of DNA damage

**Sirt1:** homolog of the yeast Sir2 protein, involved in regulating epigenetic gene silencing and suppressing recombination of rDNA.

**SMARCAL1 (SWI/SNF-related matrix-associated actin-dependent regulator of chromatin subfamily A-like):** protein that converts RPA-bound single-strand DNA into double-stranded DNA, an enzyme activity termed “annealing helicase”

**Stress induced premature senescence (SIPS):** a cellular state (see senescence) in which cells, regardless of telomere length, stop to proliferate under various stressful pathophysiological stimuli

**Structure-specific endonuclease complexes:** endonuclease complexes required for repair of specific types of DNA lesions and critical for cellular responses to replication fork failure

**Telomere:** region of repetitive nucleotide sequences at each end of a chromosome, which protects the end of the chromosome from deterioration or from fusion with neighboring chromosomes

**TIA-1:** member of an RNA-binding protein family that possesses nucleolytic activity against cytotoxic lymphocyte (CTL) target cells. The major granule-associated species is a 15-kDa protein

**Tripartite spatial organization:** refers to compartmentalization of nucleolus into the Fibrillar Centre (FC) surrounded by the Dense Fibrillar Component (DFC) and the Granular Component (GC). Polymerase-I mediated rDNA transcription occurs mainly at the interface between FC and DFC. The processing and modifications of pre-RNA and ribosome assembly begins in the DFC and is completed in the GC.

**Tumor suppressor:** group of genes that encode proteins normally acting to inhibit tumor promotion

**Unfolded protein response (ER) (UPR^ER^):** a homeostatic signaling network that orchestrates the recovery of endoplasmic reticulum (ER) function upon proteotoxic ER stress

**Unfolded protein response (mitochondria) (UPR^MT^):** a homeostatic signaling network that orchestrates the recovery of mitochondria (MT) function upon mitochondrial proteotoxic stress

**Wee1:** a nuclear tyrosine kinase belonging to the Ser/Thr family of protein kinases. Phosphorylates and inactivates cyclin B1-complexed CDK1. Acts as a negative regulator of entry into mitosis (G2 to M transition) by protecting the nucleus from cytoplasmically activated cyclin B1-complexed CDK1 before the onset of mitosis

**WRN (Werner syndrome ATP-dependent helicase):** member of the RecQ Helicase family that possesses both 3′ → 5′ helicase and 3′ → 5′ exonuclease activities. WRN is important in the maintenance of genome stability, DNA repair, replication, transcription and telomere maintenance. Mutations in *WRN* cause Werner syndrome characterized by premature aging and elevated risk of cancer

**ZRANB3:** protein possessing DNA annealing helicase and endonuclease properties, required to maintain genome stability at stalled or collapsed replication forks by facilitating fork restart and limiting inappropriate recombination that could occur during template switching events

**List of abbreviations used in the main text**

8-oxo-G: 8-Oxoguanine;

53BP1 (TP53BP1): p53 binding protein 1;

ALP: autophagy-lysosome pathway;

alt-NHEJ: alternative non-homologous end joining;

AMPK: 5’ AMP-activated protein kinase;

APC: Anaphase promoting complex;

APE1/APE2: apurinic/apyrimidic endonucleases;

APOBEC**:** apolipoprotein B mRNA editing enzyme, catalytic subunit;

ARF: alternative reading frame;

Atg: autophagy related proteins;

ATM: ataxia Telangiectasia Mutated;

ATP: adenosine triphosphate;

ATR: ataxia Telangiectasia and Rad3-related;

BACH1: BTB domain and CNC homolog 1;

BASC complex**:** BRCA1-associated genome surveillance complex;

BER: base excision repair;

BIR: break induced replication;

BLM: Bloom syndrome protein;

BRCA1: breast cancer susceptibility gene 1;

BRCA2: breast cancer susceptibility gene 2;

BRIP1: BRCA1 interacting protein C-terminal helicase 1;

Cdc6: cell division cycle 6;

CDC20: cell division cycle 20;

CDC25A: cell division cycle 25A;

CDC25B: cell division cycle 25B;

CDK: cyclin-dependent kinase;

Cdt1: chromatin licensing and DNA replication factor 1;

CFS: common fragile sites;

cGAS: cyclic GMP-AMP synthase;

CHD3.1: chromodomain-helicase-DNA-binding protein;

CHFR: checkpoint with forkhead and ring finger domain;

Chk1: checkpoint kinase 1;

Chk2: checkpoint kinase 2;

CIN: chromosomal instability;

CLs: cross-links;

CMA: chaperone mediated autophagy;

CS recombination: class switch recombination;

CSA: Cockayne Syndrome Group A;

CSB: Cockayne Syndrome Group B;

CtIP: CTBP-interacting protein;

DDR: DNA damage response;

DDR/R: DNA damage response and repair machinery;

DDT: DNA damage tolerance;

DEB: Diepoxybutane;

DFC: dense fibrillar component;

DNAM-1/CD226: DNAX accessory molecule 1;

DNA-PK: DNA-dependent protein kinase, catalytic subunit;

dNTPs: deoxynucleotide triphosphates;

DR: direct repair;

DSB(s): double strand break(s);

DUBs: deubiquitinating enzymes;

E2F1: E2F Transcription factor 1;

EGFR: epidermal growth factor receptor;

EME1: essential meiotic structure-specific endonuclease 1;

EMT: epithelial to mesenchymal transition;

EPM: enzymatic protein modifications;

eNoSC: energy-dependent nucleolar silencing complex;

ERAD: endoplasmic reticulum associated protein degradation;

ERCC1 (XPF): excision repair cross-complementation group 1;

ERCC5 (XPG): excision repair 5, endonuclease;

EXO I: exonuclease I;

FA: Fanconi anemia;

FAAP24: Fanconi anemia-associated protein of 24 kDa;

FANCC: Fanconi anemia complementation group C;

FANCD2: Fanconi anemia complementation group D2;

FANCI: Fanconi anemia complementation group I;

FANCM: Fanconi anemia complementation group M;

FC: fibrillar center;

FATC domain: FRAP, ATM, TRRAP C-terminal;

FDA: Food and Drug Administration;

FHIT: fragile histidine triad;

Gadd45α*:* growth arrest and DNA damage inducible 45α;

GC: granular component;

GEN1: Gen endonulcease homolog 1;

GI: genomic instability;

HDAC6: histone deacetylase 6;

Hh pathway: hedgehog pathway;

Hif-1: hypoxia-inducible factor-1;

HP1: heterochromatin protein 1;

HRR: homologous recombination repair;

Hsps: heat shock proteins;

Hsp90: heat shock protein 90;

ICAM1: intracellular adhesion molecule 1;

ICL: interstrand crosslink;

IκB kinase (IKK): inhibitor of nuclear factor kappa-B kinase;

IKK: inhibitor of nuclear factor kappa-B kinase;

IL-1α: interleukin 1α;

KAP1: KRAB-associated protein 1;

Ku70: Lupus Ku autoantigen protein p70;

LIG: ligase;

MAD2: mitotic arrest deficient 2;

MAPK: mitogen-activated protein kinases;

MDC1: mediator of DNA-damage checkpoint 1;

MDM2: murine double minute 2;

MGTM: O-6-methylguanine-DNA methyltransferase;

MLH1/3: mutL homolog 1/3;

MMC: Mitomycin C;

MMEJ: microhomology-mediated end-joining;

MMR: mismatch repair;

MRE11: meiotic recombination 11;

MRN: MRE11-RAD50-NBS1 complex;

MSH2/3/6: mutS homolog 2/3/6;

mtDNA: mitochondrial DNA;

MTH1: mutT homologue 1;

mtSSB: mitochondrial single-strand DNA binding protein;

MUS81: Methyl methanesulfonate and UV sensitive clone 81;

NAD: nicotinamide adenine dinucleotide;

NBS1: Nijmegen breakage syndrome 1 (Nibrin);

NEMO: nuclear factor kappa B essential modulator [also known as IKKγ (Inhibitor of nuclear factor kappa-B kinase subunit gamma)];

NEPMs: non-enzymatic protein modifications;

NER: nucleotide excision repair;

NF-κB: nuclear factor kappa-light-chain-enhancer of activated B cells;

NHEJ: non-homologous end joining;

NKG2D: natural killer group 2, member D;

NoRC: nucleolar remodeling complex;

NORs: nucleolar organizing regions;

Nrf2: nuclear factor erythroid 2 (NFE2)-related factor 2;

NuRD: nucleosome remodeling and deacetylation;

OMMAD: outer mitochondrial membrane-associated degradation;

P38MAPK: p38 mitogen activated protein kinase;

PALB2: partner and localizer of BRCA2;

PARP: poly [ADP-ribose] polymerase;

PCNA: proliferating cell nuclear antigen;

PDR: protein damage response;

PGC1-a: peroxisome proliferator-activated receptor gamma coactivator 1-alpha;

PI3K: phosphatidylinositol 3-kinase;

Pif1: petite integration frequency 1;

Plk1: polo-like kinase 1;

PMS2: PMS1 homolog 2, mismatch repair protein;

PN: proteostasis network;

PP2A: protein phosphatase 2 catalytic subunit alpha;

PrimPol: primase-polymerase;

PTM: post-translational modifications;

Rb: Retinoblastoma protein;

RBPs: RNA binding proteins;

rDNA: ribosomal DNA;

RENT: regulator of nucleolar silencing and telophase exit;

RFB: replication fork barrier;

RNF8: ring finger protein 8;

RNF168: ring finger protein 168;

RNS: reactive nitrogen species;

ROS: reactive oxygen species;

RPA: replication protein A;

RS: replication stress;

RT: replication timing;

SAC: spindle assembly checkpoint;

SASP: senescence associated secretory phenotype;

SCF: SKP, Cullin, F-box containing complex;

Sirt1: sirtuin (silent mating type information regulation 2 homolog) 1 (S. cerevisiae);

SLX1: SLX1 Structure-specific endonuclease subunit;

SLX4: SLX4 Structure-specific endonuclease subunit;

SMARCAL1: SWI/SNF-related matrix-associated actin-dependent regulator of chromatin subfamily A-like;

SNSs: single nucleotide substitutions;

SSA: single-strand annealing;

SSB: single-strand break;

SR: stress response;

SRP: stress response pathways;

STING: stimulator of interferon genes;

TFIIH: transcription factor II human;

TFAM: mitochondrial transcription factor A;

TIGAR: TP53-induced glycolysis and apoptosis regulator;

TLS: translesion synthesis repair;

TS: template switching;

UPP: ubiquitin-proteasome;

UPR^ER^: unfolded protein response in the endoplasmic reticulum;

UPR^MT^: mitochondrial unfolded protein response;

USP11: ubiquitin specific peptidase 11;

USP44: ubiquitin specific peptidase 44;

VHL: Von Hippel Lindau;

Wee1: WEE1 G2 checkpoint kinase;

Wip1: also called protein phosphatase Mg^2+^/Mn^2+^ dependent 1D (PPM1D);

Wnt*:* Wnt family and int;

WRN: Werner syndrome helicase;

WWOX: WW domain-containing oxidoreductase;

XPA: Xeroderma pigmentosum group A-complementing protein;

XPC: Xeroderma pigmentosum, complementation group C;

XPE (DDB2): Xeroderma pigmentosum complementation group E protein;

XRCC4: X-ray repair complementing defective repair in Chinese hamster cells 4;

XRCC5/Ku80: X-ray repair complementing defective repair in Chinese hamster cells 5;

XRCC6/Ku70: X-ray repair complementing defective repair in Chinese hamster cells 6
