# Supplementary figures and images for: Integrating the DNA damage and protein stress responses during cancer development and treatment
Source: J Pathol. 2018 Jul 19;246(1):12–40. doi: 10.1002/path.5097 (PMC6120562; doi:10.1002/path.5097)

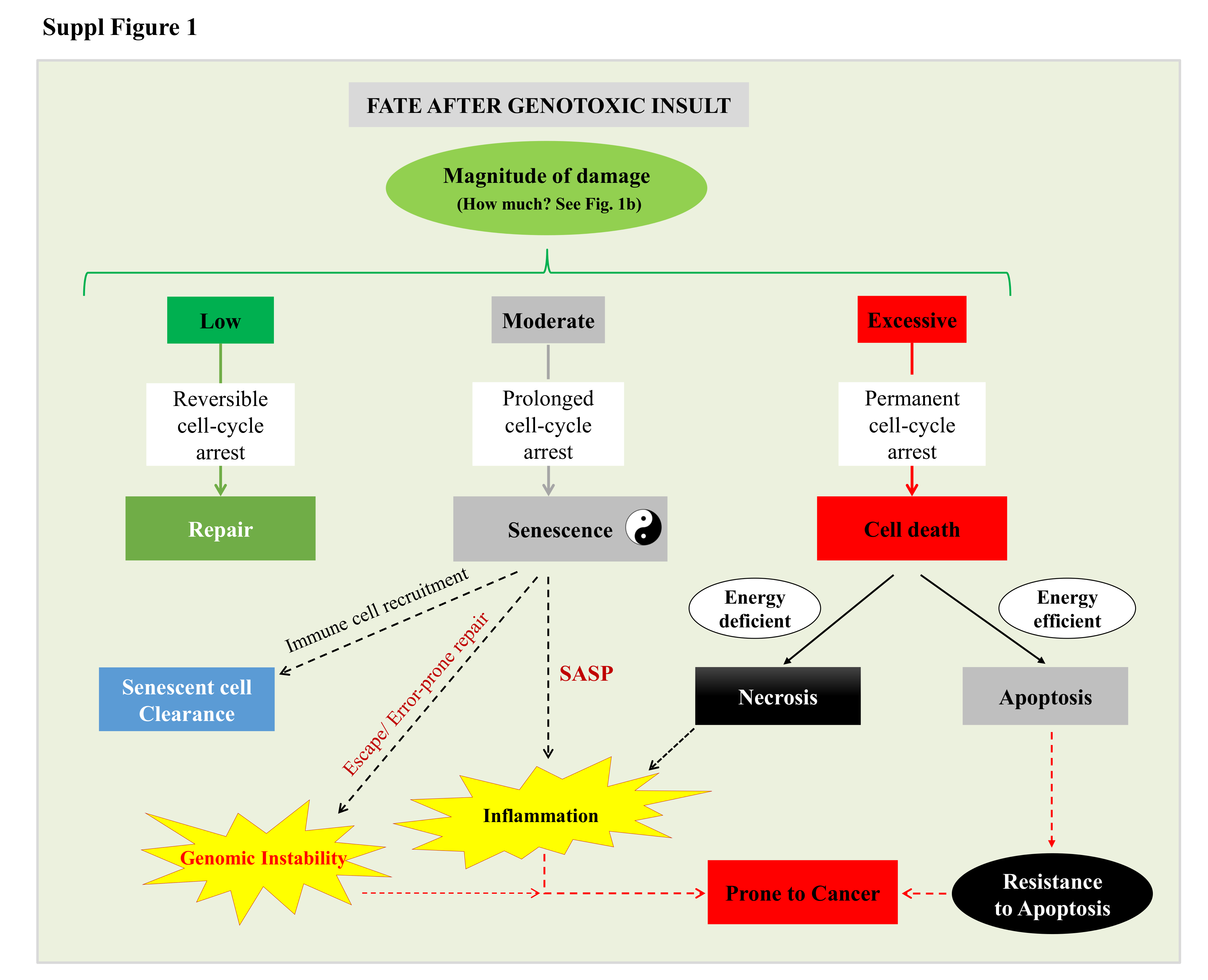

Supplement: Supplementary file 4 — Figure S1. The cellular fate following genotoxic insults. The magnitude of the genotoxic insult (low, moderate, excessive) determines cells fate (effective repair, senescence or cell‐death, respectively). Under certain conditions, determined by the stress response parameters (Figure 1B), senescence can present a “dark side”. Likewise, necrosis and/or resistance to apoptosis can build up a pro‐tumorigenic environment (see text for details and references). SASP: senescence‐associated secretory phenotype. [file PATH-246-12-s004.tif]

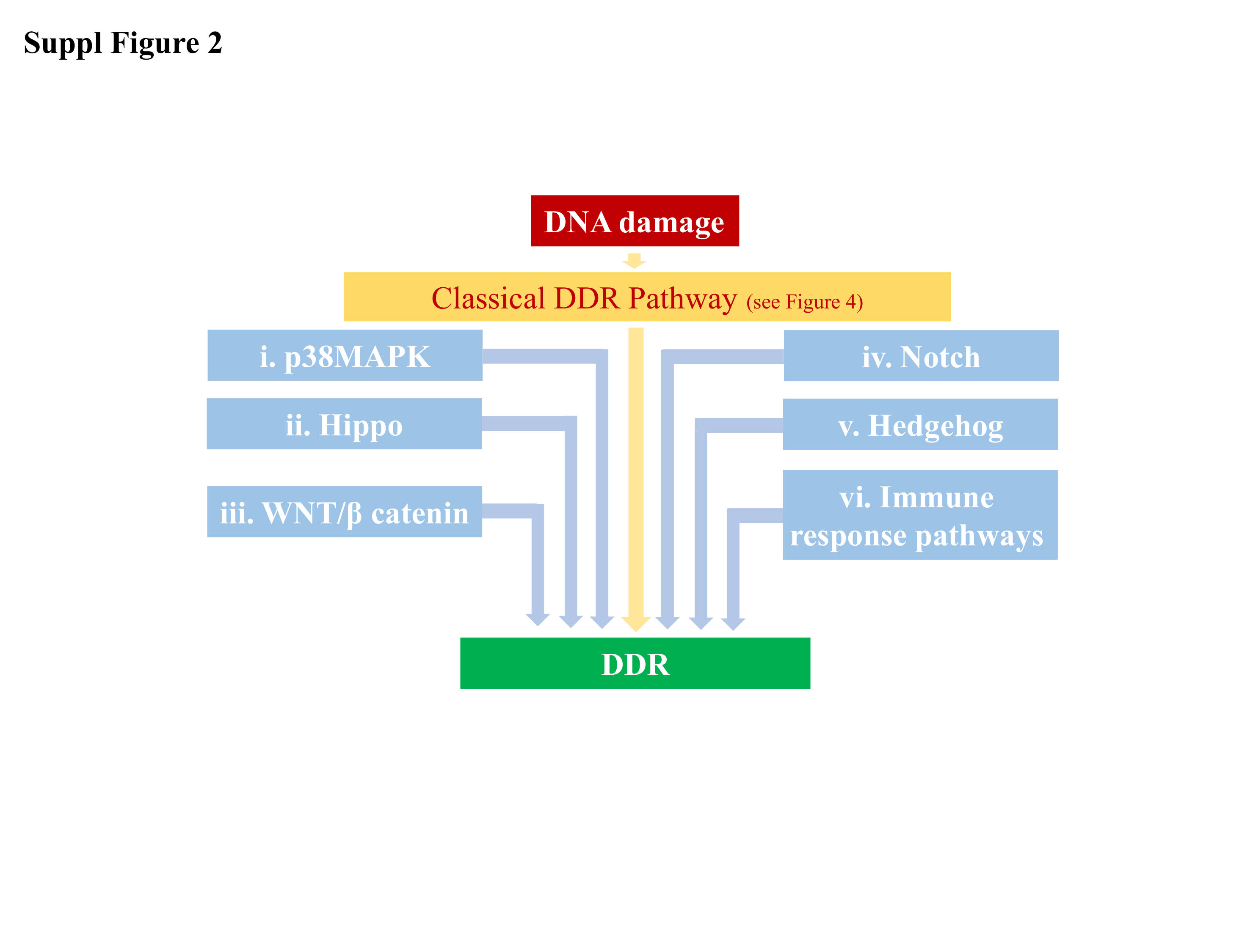

Supplement: Supplementary file 5 — Figure S2. Other pathways that contribute to DDR signaling. Accumulating data demonstrate that the DDR function is complemented and/or it cross‐talks with other signaling routes, which also respond to DNA damage 420, 421, 422. To what extent these signaling pathways modulate the DDR function is a subject that has not been fully elucidated. Nevertheless, the implementation of multiple signaling cascades in a DDR network highlights the need for the DNA damage machinery to detect and respond to a wide range of stimuli in various cellular scenarios, underscoring the highly modular organization of the DDR 422. (i) One such signaling pathway involved in DDR is the p38 MAPK. It is one of the three main groups of mitogen‐activated protein kinases (MAPK). It contributes in the G2/M checkpoint, to facilitate DNA repair, via three possible routes: a) the direct phosphorylation of p53, which results in the dissociation of p53 from Mdm2 thus preventing p53 ubiquitination and degradation, b) the association with Gadd45α, which interacts with p53 and increases its stability, and c) the phosphorylation and inhibition of the phosphatase Cdc25B which is responsible for driving the cell cycle through activation of the Cyclin B/Cdc2 complex 53, 423. In addition, p38 MAPK activation can induce G1/S checkpoint in response to a variety of cellular stresses such as osmotic shock or cellular senescence 53, 423. (ii) Hippo signaling pathway is also implicated in the DDR. Further to a wide spectrum of cellular roles, components of the Hippo pathway cooperate with central orchestrators of the DDR, namely the ATR‐Chk1 and ATM‐Chk2 signaling nodes 424, 425. (iii) Wnt/ β catenin pathway, which has important functions in controlling gene expression, cell polarity and adhesion, is also involved in the repair of DNA damage specifically due to oxidative stress, through interaction with DDR at different levels 426, 427, 428. (iv) NOTCH pathway is a highly conserved signaling system that functions in d [file PATH-246-12-s006.tif]

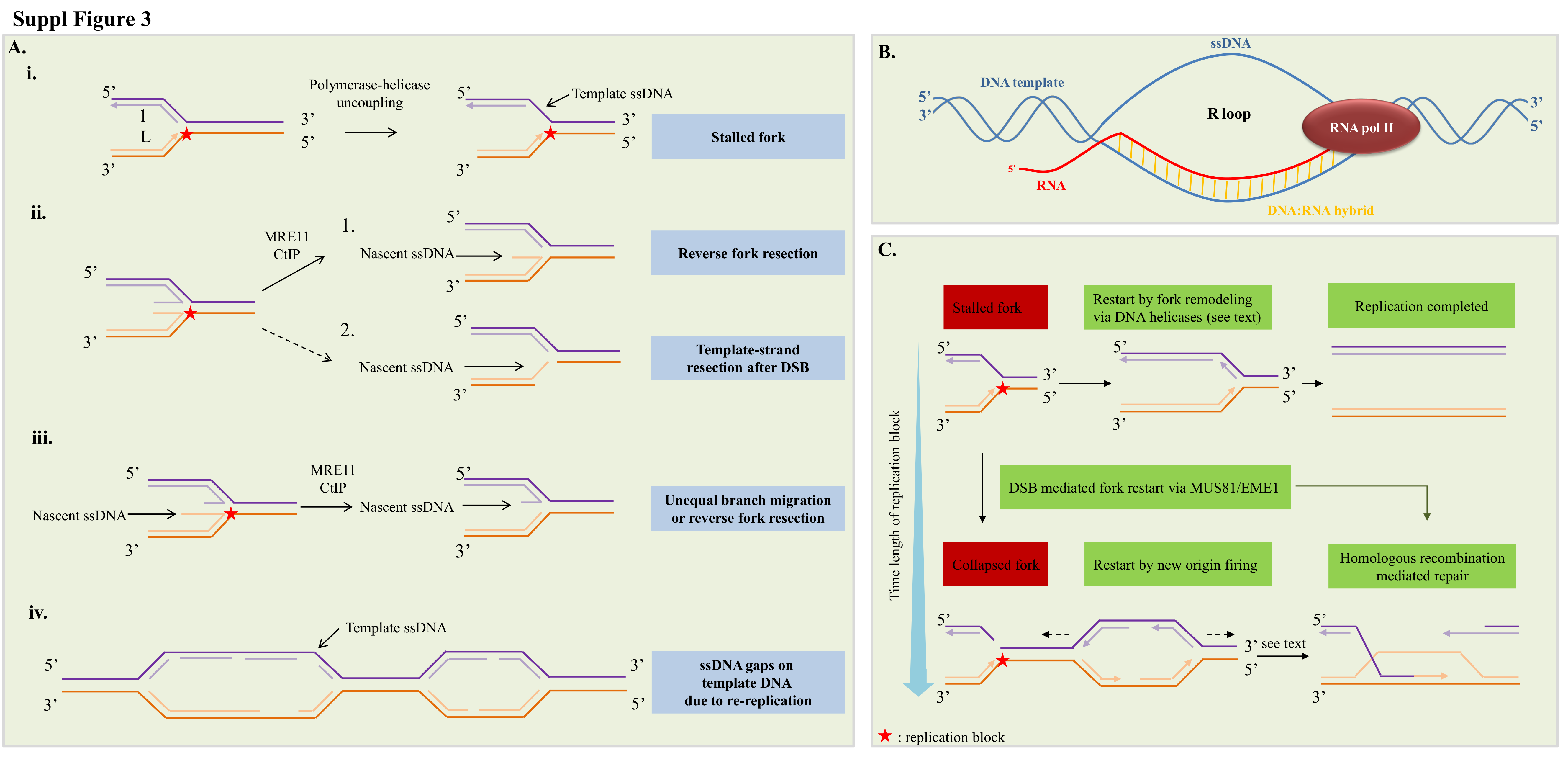

Supplement: Supplementary file 6 — Figure S3. Replication‐transcription intermediates and replication fork restart. (A) Replication intermediate lesions harboring single stranded DNA (ssDNA). (i) Uncoupling of the replicative helicase and polymerases results in generation of ssDNA due to excessive unwinding of the template (stalled fork). (L: leading strand; l: lagging strand) (ii) A stalled replication fork may undergo remodeling by creating an intermediate reverse fork also known as “chicken foot” structure: (ii‐1) Direct CtIP processing of the reversed fork may lead to nascent strand ssDNA formation. (ii‐2) Cleavage by SLX4‐docking nucleases generates DNA double strand break that is subsequently followed by resection resulting into nascent strand ssDNA generation. (iii) Unequal branch migration or resection (by CtIP) of a reversed fork can also lead to generation of template ssDNA. (iv) Deregulated firing of clustered origins leads to replication stress and accumulation of gaps in the nascent strands, leaving template ssDNA (see text for details and references) (B) Transcription intermediates. R loops are the predominant transcription generated intermediates and represent a three‐stranded nucleic acid structure that comprises two branches, an RNA–DNA hybrid and an ssDNA. The former can impede completion of replication leading to replication fork stalling, collapse and DSBs formation, while the latter can serve as a substrate to DNA damaging agents and cellular enzymes [APOBEC deaminases (Table 1)] resulting in DNA lesions and/or nicks (see text for details and references). (C) Restart of stalled or collapsed replication forks. Depending on the duration (how long) of the replication block, forks can stall or collapse. Restart of stalled forks is promoted by fork remodeling factors, while collapsed forks rely on DSB mediated restart through homologous recombination repair, whereas new origins are concurrently fired (see text for details and references). [file PATH-246-12-s001.tif]

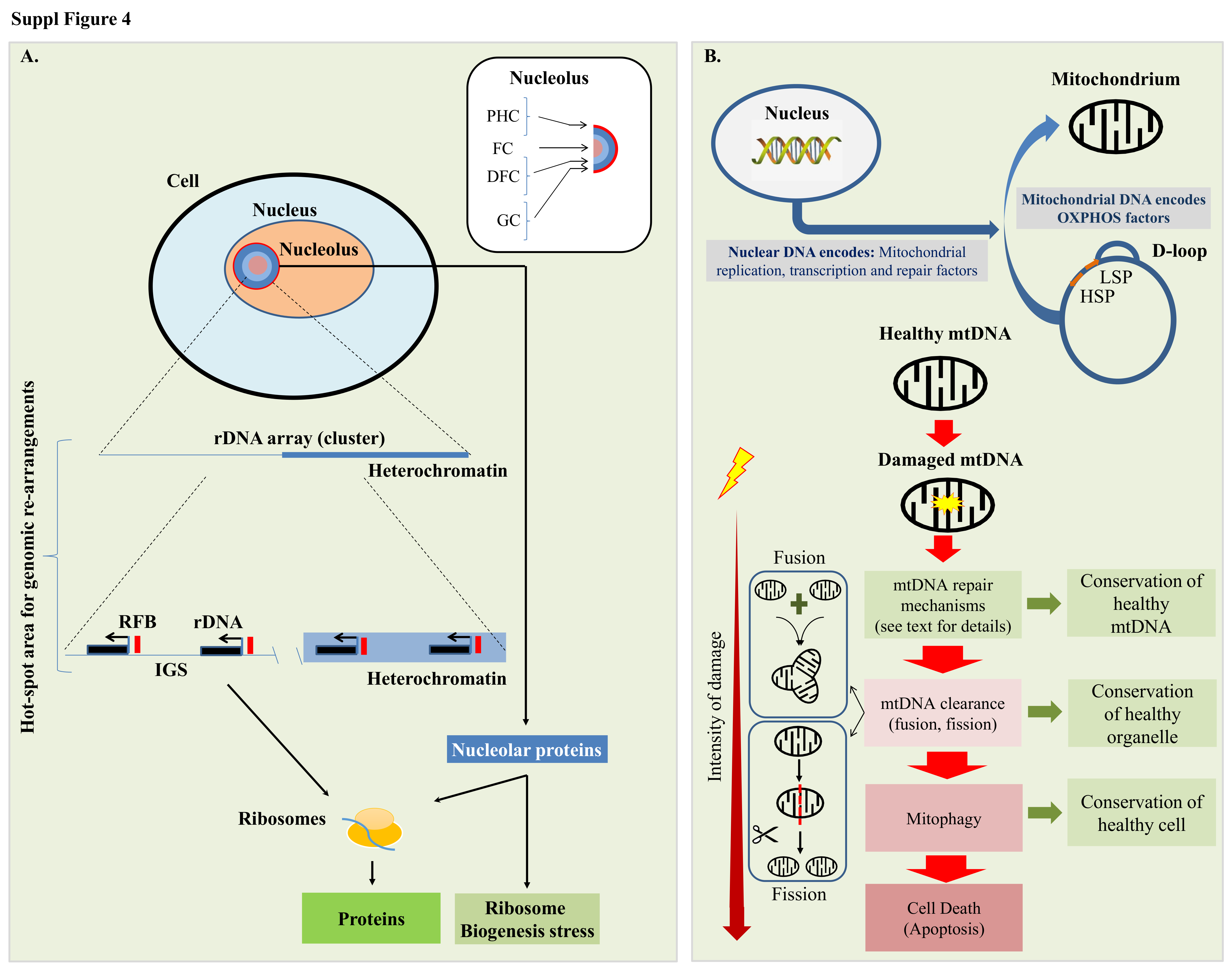

Supplement: Supplementary file 7 — Figure S4. (A) Nucleolus and rDNA organization. Schematic representation of the rDNA repeats, an organization that renders them susceptible to replication‐transcription collisions (see text for details). PHC: Perinuclear Heterochromatin, FC: Fibrillar Centre, DFC: Dense Fibrillar Component and CC: Granular Component (see text for details and references). (B) Maintaining mitochondrial DNA integrity: Nuclear and mitochondrial DNAs are interdependent. Cartoon of the mitochondrial DNA: D (Displacement)‐loop: a short nucleotide segment complementary to the light (L)‐strand that displaces the heavy (H)‐strand of the mitochondrial DNA. It contains promoters (LSP and HSP) for the RNA transcription from the two strands (heavy and light, respectively) of mitochondrial DNA, possibly involved in the organization of the mitochondrial nucleoid (see text for details and references); LSP: Light strand promoter. The promoter is responsible for gene transcription from the light strand (lower molecular mass) of mitochondrial DNA; HSP: Heavy strand promoter. The promoter is responsible for gene transcription from the heavy strand (higher molecular mass) of mitochondrial DNA. Depending on the magnitude of the mitochondrial DNA damage three levels of repair may take place (see text for details and references). [file PATH-246-12-s002.tif]

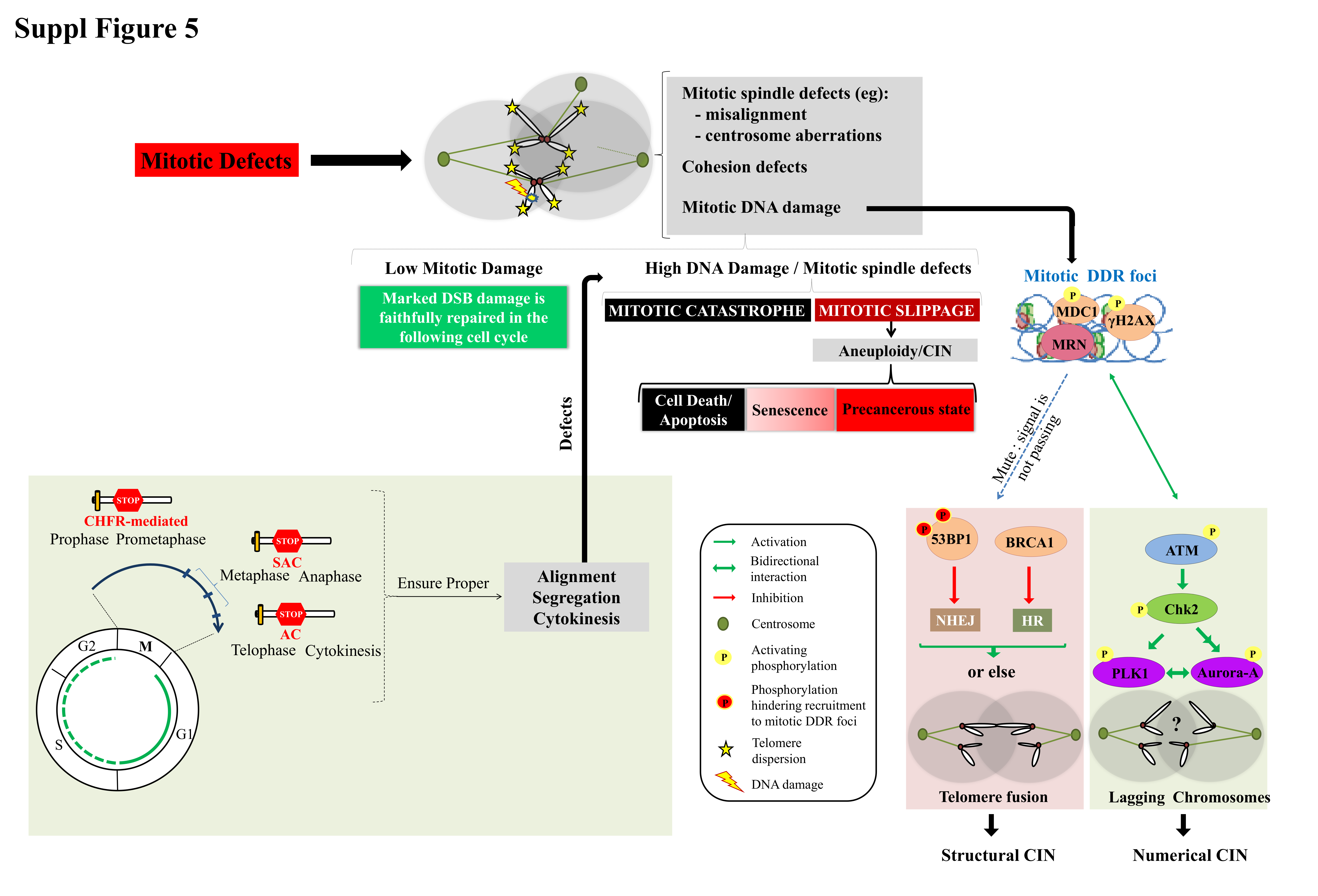

Supplement: Supplementary file 8 — Figure S5. Monitoring Mitosis (DDR surveillance). During M phase checkpoints monitor the proper alignment, segregation and cytokinesis (see lower left panel). In response to a mitotic defect, such as misalignment and/or DSBs, cell fate depends on the context (i.e. p53 status) and the extent of the damage (how much): i) low mitotic damage is marked, and repaired in the subsequent cell cycle in the daughter cells (continuous green line corresponds to G1 phase where the majority of DNA lesions are repaired, however, mitotic DNA lesions can also be repaired in S and G2 phase depicted by the dashed green line‐see lower left cell cycle panel), ii) high DNA damage or mitotic spindle defects may lead to mitotic catastrophe or mitotic slippage, which in turn generates aneuploidy and/or CIN. The later can lead to cell death, senescence or development of precancerous lesions. Upon induction of DSBs during mitosis, MRN, and phosphorylated MDC1 and Η2ΑΧ are recruited to the damaged site forming the mitotic DDR foci (see right panel). Notably, 53BP1 and BRCA1 are not recruited to the site of damage blocking NHEJ and HR activation, respectively, preventing telomere fusion (mute DDR). An adverse outcome of mitotic DDR activation is kinetochore‐microtubule stabilization mediated by activation of PLK1 and Aurora kinase A that in turn promotes merotelic attachment and the formation of lagging chromosomes resulting in numerical CIN. However, it is not yet clear under what circumstances activation of mitotic DDR leads to this unfavorable outcome, instead of marking the DNA damage and proceeding to repair in the following cell cycle (marked with a question mark; right lower panel). “P” within red colored circles depicts the two phosphorylation sites of 53BP1 at Threonine‐1609 and Threonine‐1618 that prevent it from recruitment to DDR foci. CIN: chromosomal instability [file PATH-246-12-s007.tif]

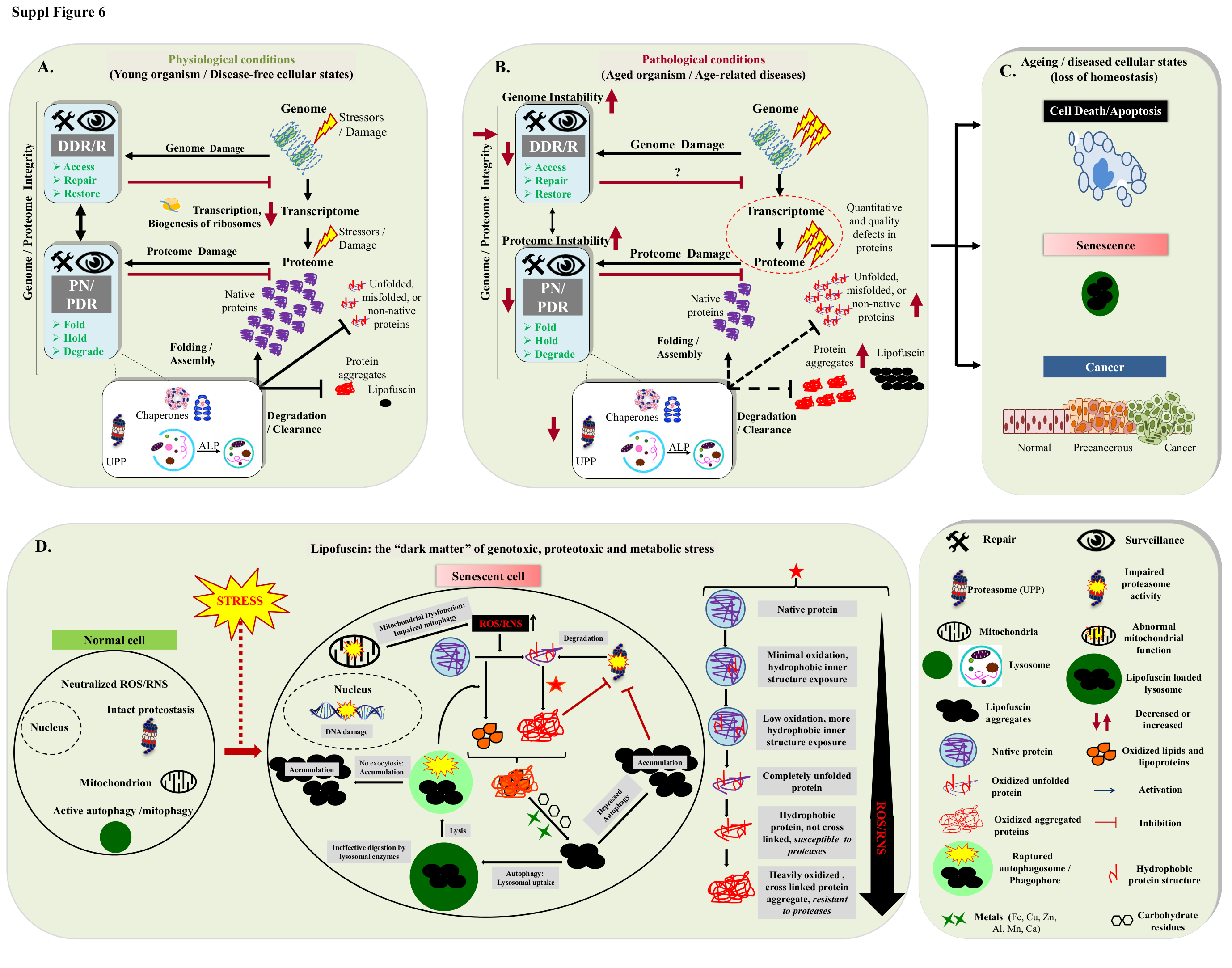

Supplement: Supplementary file 9 — Figure S6. Functional interplay and interdependence of genome and proteome maintenance modules (DDR and PDR surveillance). (A) The PN along with PDR are actively involved in DDR efficiency since by assuring proteome integrity they maintain the functionality of the protein machines that safeguard genome stability. On the other hand, DDR induces a number of proteostatic and/or metabolic adaptations, including suppression of transcription and ribosomal biogenesis, indicating the functional interdependence of the two pathways. These pathways are fully active in young organisms. (B‐C) The age‐related collapse of proteostatic modules functionality and/or expression levels (B) results in the gradual accumulation of non‐functional polypeptides, protein aggregates or lipofuscin, compromising proteome integrity and leading to genomic instability (and thus increased chances for carcinogenesis) as a result of ineffective DNA maintenance and/or repair. Eventually, a vicious cycle may form where a mildly unstable genome accelerates proteome instability due to synthesis of mutated polypeptides that progressively increase the attrition of protein machines resulting in an increasingly stressful cellular landscape that favors the appearance (C) of cellular senescence, cell death or age‐related diseases (e.g. cancer). (D) In normal cells, production of ROS or RNS is neutralized by anti‐oxidant responses while intact PN ensures normal protein turnover. During stress induced premature senescence (Glossary) or in aged tissues the levels of ROS/RNS increase leading to lipid and protein oxidation in the cytoplasm. As this process evolves, oxidized proteins become unfolded and intra‐ and/or inter‐molecular cross links occur, forming non‐degradable oxidized protein aggregates; the latter along with oxidized lipids/lipoproteins, carbohydrate residues and metals form undegradable lipofuscin which accumulates mainly in lysosomes, while only a minor amount is found free in the cytosol. Cytosolic [file PATH-246-12-s003.tif]
