# Supplementary material for: Integrating the DNA damage and protein stress responses during cancer development and treatment
Source: J Pathol. 2018 Jul 19;246(1):12–40. doi: 10.1002/path.5097 (PMC6120562; doi:10.1002/path.5097)
Supplement: Supplementary file 10 — Table S1. Representative categories and types of human RNAs involved in physiological processes and diseases, including cancer [file PATH-246-12-s010.docx]

**Table S1**. Representative categories and types of human RNAs^*^ involved in physiological processes and diseases, including cancer

Reference numbers refer to the main text list

| **lncRNA^1^** | **RNAs > 200 nucleotides** | | |
| --- | --- | --- | --- |
| **Name** | **Phenotype/function** | **Proposed mechanism** | **Refs** |
| *XIST* | Required for transcriptional silencing of one of the X chromosome during development in female mammals. | *XIST* coats the X-chromosome, modifying chromosome structure and spreading to newly accessible locations | 432 |
| *ANRIL* | Interacts with CBX7 of the PRC1 promoting H3K27 methylation. | Elevated levels in non-small cell lung carcinoma, gastric cancer, hepatocellular cancer, bladder cancer | 433 |
| *HOTAIR* | Interacts with PRC2 and LSD1/CoREST/ REST complexes leading to H3K27 methylation and H3K4 demethylation and ultimately gene silencing. | Up regulated in breast, hepatocellular, gastric, colorectal, pancreatic cancer | 433 |
| *HOTTIP* | Interacts with WDR5/MLL complex leading to activation of HOXA genes. | Up regulated in hepatocellular carcinoma | 433 |
| *CCAT1-L* | Favors chromatin looping and mediates interaction of CTCF at Myc promoter region. | Up regulated in colorectal cancer | 433 |
| *TERC* | TERC associates with TCAB1 (WDR79) during telomerase assembly. | Up regulated in early esophageal cancer and gastric cancer | 433 |
| *PTENP1* | Decoys miRNAs that target 3′UTR of coding PTEN. | Down regulated in hepatocellular carcinoma and renal cell carcinoma | 433 |
| *Linc ROR* | Binds miR-145 leading to inhibition of OCT4, NANOG and SOX2 degradation. | Up regulated in breast cancer | 433 |
| *MALAT1* | Binds miR-124 and determines  GRB2 levels (target of miR-124). | Up regulated in lung adenocarcinoma, esophageal and bladder cancer | 433 |
| *ANRASSF1* | Forms a DNA:RNA hybrid and recruits PRC2 to *RASSF1A* locus. | Up regulated in breast cancer | 433 |
| *lncRNA-ATB* | 1. Binds IL-11 prolonging its translational availability.  2. Binds miR200 family leading to upregulation of ZEB1 and ZEB2. | 1. Up regulated in hepatocellular carcinoma  2. Induces mesenchymal morphological features and promotes cell invasion | 433 |
| *HULC* | Binds miR-372 leading to decreased repression of PRKACB. | Up regulated in hepatocellular carcinoma | 433 |
| *KCNQ1OT1/KvLQT1AS/*  *LIT1* | Affects the imprinting status of the centromeric region of 11p15.5.  Suppresses paternal allele expression of the *KCNQ1OT1* and *CDKN1C* genes. | Affected in lung, prostate and colorectal cancer | 433  434  435 |
| *PACER* | Promotes COX2 expression. | Interacts with NF-kB p50 subunit,  prevents it from binding to and repressing  COX2 | 436 |
| *lnc-DC* | Highly expressed in all DC populations.  Promotes CD40, CD80, HLA-DR expression on DCs. | Blocks dephosphorylation of STAT3 | 436 |
| *HOTAIRM1* | Knockdown results in reduced CD11B and CD18 expression.  Reduced granulocytic differentiation of cell lines. | Activated downstream of RA signaling;  mechanism unknown | 436 |
| *NKILA* | Induced in response to TNF and IL-1b to regulate NF-kB signaling in breast cancer cell lines. | Blocks IkB phosphorylation | 436 |
| *NEAT1* | Regulates IL-8 synthesis following immune stimulation. | Alters localization of the repressor protein  SFPQ | 436 |
| *lncRHOXF1* | Suppresses the expression of viral response genes in trophoblast progenitors. | Unknown | 436 |
| **small RNAs** | **RNAs ~ 20-35 nucleotides** | | |
| **micro RNAs (miRNAs)^2^** | | | |
| let-7 | 1. Involved in breast, prostate, hepatocellular, gastric, lung cancer.  2. Involved in colon, pancreatic, hepatocellular cancer. | Down regulated  Up regulated | 437 438 |
| miR-10b | Involved in breast cancer and glioblastoma | Up regulated | 437438 |
| miR-17-5p | Involved in breast cancer and neuroblastoma. | Down and up regulated respectively | 437 438 |
| miR-18 | Involved in hepatocellular carcinoma. | Up regulated | 437 438 |
| miR-21 | 1. Involved in breast, colorectal ovarian, hepatocellular, cervical, pancreatic, lung cancers.  2. Involved in pituitary adenomas  3. Involved in granulocyte progenitors and monoblast maturation. | Up regulated  Down regulated  Down regulated | 437 438 |
| miR-23a | Involved in pancreatic cancer. | Up regulated | 437 438 |
| miR-23b | Involved in uterine leiomyoma. | Up regulated | 437 438 |
| miR-23 | Involved in hepatocellular carcinoma. | Up regulated | 437 438 |
| miR-34 | 1. Involved in lung, pancreatic cancers and neuroblastomas.  2. Involved in renal, colon, hepatocellular carcinomas. | Down regulated  Up regulated | 437 438 |
| miR-150 | Involved in megakaryocyte and mature B cells differentiation. | Down regulates c-MYB | 438 |
| miR-155 | Involved in macrophage generation and naïve T cells maturation. | Up regulation inhibits FADD, TNFR, RipK1, c-MAF | 438 |
| miR-133 | Involved in heart fibrosis, cellular viability and electrical remodeling. | Down regulated | 438 |
| miR-208b | Involved in heart hypertrophy. | Up regulation inhibits THRAP1 | 438 |
| miR-24 | Involved in myotube formation. | Up regulated | 438 |
| miR-181 | Involved in myogenesis. | Up regulation inhibits HOXA11 | 438 |
| miR-221/ 222 | Involved in cell cycle exit of myogenic stem cells. | Down regulation leads to p27^KIP1^ up regulation | 438 |
| miR-124 | Involved in brain development. | Up regulated | 438 |
| miR-134 | Involved in brain development. | Up regulated | 438 |
| miR-29b | Involved in Alzheimer’s disease. | Down regulated, releasing suppression of BACE1 | 438 |
| miR-107 | Involved in Alzheimer’s disease. | Down regulated, releasing suppression of BACE1 | 438 |
| miR-183 family | Involved in inner ear development. | Formation of the stereocilia of hair cells | 438 |
| **small nucleolar (snoRNAs)^3^** | | | |
| SNORD112-1 | Involved in Acute Myeloid Leukemia. | Up regulated | 439 |
| U50 | Involved in breast and prostate cancer. | Down regulated | 440 |
| h5sn2 | Involved in meningioma. | Down regulated | 440 |
| RNU43 | Involved in breast cancer and HNSCC. | Down regulated | 440 |
| RNU44 | Involved in breast cancer and HNSCC. | Down regulated | 440 |
| snoRD33 | Involved in NSCLC. | Up regulated | 440 |
| snoRD66 | Involved in NSCLC. | Up regulated | 440 |
| snoRD76 | Involved in NSCLC. | Up regulated | 440 |
| snoRA42 | Involved in NSCLC. | Up regulated | 440 |
| snoRD44 | Involved in breast cancer. | Up regulated | 440 |
| SNORD115 | Involved in neurodegenerative diseases of Prader - Willi syndrome. | Impacts on 5-*HT2CR* serotonin receptor mRNA level in brain | 439 |
| SNORD116 | Involved in neurodegenerative diseases of Prader - Willi syndrome. | Impacts on 5-*HT2CR* serotonin receptor mRNA level in brain | 439 |
| v-snoRNA1 | Found in B lymphocytes infected with Epstein-Barr virus. | Interacts with *BALF5* mRNA and induces specific degradation of target mRNA | 439 |
| U38 | Found in serum of patients with anterior cruciate ligament (ACL) injury. | Up regulated | 439 |
| U48 | Found in serum of patients with anterior cruciate ligament (ACL) injury. | Up regulated | 439 |
| snoRA15 | X-linked dyskeratosis congenital. | Down regulated | 441 |
| **piwi-interacting RNAs (piRNAs)^4,5,6^** | | | |
| piR-4987 | Involved in breast cancer. | Up regulated | 26 |
| piR-20365 | Involved in breast cancer. | Up regulated | 26 |
| piR-34736 | Involved in breast cancer. | Down regulated | 26 |
| piR-36249 | Involved in breast cancer. | Down regulated | 26 |
| piR-823 | Involved in gastric cancer. | Down regulated | 26 |
| piR-59056 | Involved in gastric cancer. | Up regulated | 26 |
| piR-32105 | Involved in gastric cancer. | Up regulated | 26 |
| piR-823 | Involved in multiple myeloma. | Up regulated | 26 |
| piR-017061 | Involved in pancreatic cancer. | Down regulated | 26 |
| piR-L-163 | Involved in lung cancer. | Down regulated | 26 |
| **circular RNA^7^** | **Circularized transcripts, byproducts from imperfect splicing** | | |
| cANRIL | Associated with an increased risk of  Atherosclerosis. | Repression of the *CDKN2A* locus | 442 |
| cZNF292 | Controls angiogenesis. | Regulated by hypoxia in endothelial cells | 442 |
| ciRS-7 | Aids the clearance of amyloid peptides. | Up-regulates UBE2A, inhibits miR-7 function in islet b cells, which in turn improves insulin secretion | 442 |
| circFAM169A | Involved in bladder cancer. | Down regulated | 442 |
| cirITCH | Involved in lung cancer. | Down regulated | 442 |
| hsa_circ_002059 | Involved in gastric cancer. | Down regulated | 442 |
| circZFR | Involved in bladder cancer. | Up regulated | 442 |

* For a comprehensive description and information on each category of RNAs see:

1. <http://lncrnadb.org/>

2. <http://www.mirbase.org/>

3. <https://www-snorna.biotoul.fr/>

4. <http://regulatoryrna.org/database/piRNA/>

5. <http://pirnabank.ibab.ac.in/>

6. <https://www.bioinfo.mochsl.org.br/~rpiuco/pirna/>

7. <http://www.circbase.org/>

**Abbreviations**

*ANRASSF1:* antisense intronic non-coding RASSF1;

*ANRIL:* antisense non-coding RNA in the INK4 locus (CDKN2B-AS);

*BALF5:* DNA polymerase catalytic subunit;

*BACE1*: Beta-secretase 1;

*CBX7*: Chromobox protein homolog 7;

*CCAT1-L:* Colon cancer associated transcript 1 lncRNA;

*CDKN2A*: Cyclin-dependent kinase inhibitor 2A;

*CD11A*: Integrin subunit alpha M;

*CD18*: Integrin subunit beta 2;

*CD40*: Tumor necrosis factor receptor superfamily member 5;

*CD80*: B-lymphocyte activation antigen B7; *CDKN1C:* Cyclin-dependent kinase inhibitor 1C (p57^Kip2^);

*c-MAF*: MAF BZIP transcription factor;

*c-MYB*: MYB (myeloblastosis) proto-oncogene, transcription factor;

*COX2*: Cyclooxygenase 2;

*CTCF*: CCCTC-binding factor;

*DCs*: Dendritic cells;

*FADD*: Fas associated via death domain;

*GRB2*: Growth factor receptor bound protein 2;

*H3K27*: Histone 3 lysine 27;

*H3K4*: Histone 3 lysine 4;

*HLA-DR*: major histocompatibility complex, class II, DR;

*HOTAIR:* HOX transcript antisense RNA;

*HOTAIRM1:* HOX antisense intergenic RNA myeloid 1;

*HOTTIP:* HOXA distal transcript antisense RNA;

*HOXA*: homeobox A cluster;

*HT2CR:* 5-hydroxytryptamine receptor 2C;

*HULC:* Hepatocellular carcinoma up-regulated long non-coding RNA;

*IL*: Interleukin;

*KCNQ1OT1:* KCNQ1 opposite strand/antisense transcript 1;

*KCNQ1OT1/KvLQT1AS/LIT1:* Kcnq1 opposite transcript 1, or long QT intronic transcript 1;

*lnc-DC:* lnc-DC in dendritic cells;

*lncRHOXF1:* lncRNA from the rhox homeobox family member 2B gene on X chromosome;

*lncRNA-ATB:* Long noncoding RNA-activated by transforming growth factor β;

*LSD1/CoREST/REST*: HDAC1/lysine-specific demethylase 1 (HLCR) repressor complex;

*MALAT1:* Metastasis associated lung adenocarcinoma transcript 1;

*c-MYB*: MYB (myeloblastosis) proto-oncogene, transcription factor;

*NANOG*: Nanog homeobox (Tír na nÓg legend);

*NEAT1:* nuclear paraspeckle assembly transcript 1;

*NF-kB*: Nuclear factor kappa-light-chain-enhancer of activated B cells;

*NKILA:* NF-κB interacting lncRNA;

*OCT4*: Octamer-binding transcription factor 4;

*PACER:* P50-Associated COX-2 extragenic RNA;

*PRC1/2*: Polycomb repressive complex 1/2;

*PRKACB*: Protein kinase cAMP-activated catalytic subunit beta;

*PTEN*: Phosphatase and tensin homolog;

*PTENP1:* PTEN pseudogene-1;

*RA*: Retinoic acid;

*RASSF1A:* Ras association domain family member 1;

*RipK1*: Receptor interacting serine/threonine kinase 1;

*SFPQ*: Splicing factor proline and glutamine rich;

*SOX2*: SRY-box 2;

*STAT3*: Signal transducer and activator of transcription 3;

TCAB1 (WDR79): Telomerase cajal body protein 1, synonym of *WRAP53*;

*THRAP1*: Thyroid hormone receptor-associated protein 1, synonym of Mediator complex subunit 13 (MED13);

*TERC:* Telomerase RNA component;

*TNF*: Tumor necrosis factor;

*TNFR*: Tumor necrosis factor receptor;

*UBE2A*: Ubiquitin conjugating enzyme E2 A;

*WDR5/MLL*: WD repeat domain 5/ Myeloid/Lymphoid or Mixed-lineage leukemia protein 1;

*XIST:* X inactive specific transcript; *ZEB1/2*: Zinc finger E-box binding homeobox 1/2.
