# Supplementary material for: Integrating the DNA damage and protein stress responses during cancer development and treatment
Source: J Pathol. 2018 Jul 19;246(1):12–40. doi: 10.1002/path.5097 (PMC6120562; doi:10.1002/path.5097)
Supplement: Supplementary file 11 — Table S2. The implications of defective key components of the DNA damage response mechanisms in the pathogenesis of specific clinical syndromes in humans [file PATH-246-12-s009.docx]

**Table S2.** The implications of defective key components of the DNA damage response mechanisms in the pathogenesis of specific clinical syndromes in humans

Reference numbers refer to the main text list

| ***BER pathway [443-446]*** | | |
| --- | --- | --- |
| **Defective gene** | **Related clinical syndrome** | **Specific clinical features** |
| *OGG1* | Associated with cancer risk in *BRCA1* and *BRCA2* mutation carriers |  |
| *NTHL1* | NTHL1-associated polyposis |  |
| *MUTYH* | MUTYH-associated polyposis | Increased lifetime risk of colorectal cancer (43% to almost 100% in the absence of timely surveillance);  typically associated with ten to a few hundred colonic adenomatous polyps (evident at a mean age of about 50 years); colonic cancer develops in some individuals with biallelic MUTYH pathogenic variants in the absence of polyposis; duodenal adenomas (17%-25%), also: serrated adenomas, hyperplastic/sessile serrated polyps, and mixed (hyperplastic and adenomatous) polyps; modestly increased risk for rather late-onset malignancies of the ovary, bladder, and skin; evidence for an increased risk for breast and endometrial cancer, sebaceous gland tumors, thyroid abnormalities (multinodular goiter, single nodules and papillary thyroid cancer). |
| *PNKP* | MCSZ disorder | Microcephaly; early-onset intractable seizures and developmental delay. |
| *Pol ε* | FILS syndrome | Facial dimorphism; livedo; immunodeficiency and short stature. |
| ***NER pathway [447-450]*** | | |
| **Defective gene** | **Related clinical syndrome** | **Specific clinical features** |
| *XPA, XPB, XPC, XPD, XPE, XPF, XPG* | Xeroderma pigmentosum | Parchment skin and freckles, limited to sun-exposed areas of the skin; progressive degenerative alterations of the skin and eyes; increased (>1000-fold) risk to develop skin cancers (basal cell carcinomas and squamous cell carcinomas, less frequently melanomas); progressive neurologic abnormalities. |
| *CSA, CSB* | Cockayne’s syndrome | Cutaneous photosensitivity; skeletal abnormalities such as bird-like face, dental caries and kyphosis of the spinal cord; osteoporosis in older patients; severe early onset progressive neurological degeneration with delayed psychomotor development, gait defects and mental retardation; microcephaly; sensorineural hearing loss; pigmentary retinopathy; wizened facial appearance; thin hairs and cataracts; impaired sexual development and postnatal growth failure (cachectic dwarfism); no predisposition to develop skin cancer. |
| *XPD, XPB, TFIIH* | Trichothiodystrophy  (also included: Pollitt syndrome, Tay's syndrome, Amish brittle hair syndrome, Sabinas syndrome and Marinesco–Sjögren syndrome) | Sulfur-deficient brittle hair and ichthyosis (scaling of the skin) in combination with mental and physical retardation; photosensitivity (relatively mild and rare pigmentation abnormalities; no cutaneous malignancies have been reported) collodion baby (transparent shiny skin, also observed in some keratinization disorders); male and female hypogonadism and cryptorchidism (undescended testis); short stature. |
| ***MMR mechanism [451-453]*** | | |
| **Defective gene** | **Related clinical syndrome** | **Specific clinical features** |
| *hMLH1, hMSH2*  *Rare: hPMS1, hPMS2, hMSH6*  (Microsatellite instability  and epigenetic methylation) | Hereditary non-polyposis colorectal cancer (HNPCC) (Lynch I and II syndromes) | Lynch I: Carcinoma of the right colon (70% of colorectal cancers, proximal to the splenic flexure) + synchronous and metachronous tumours. |
|  |  | Lynch II: Similar colorectal cancer + extracolonic  cancers (endometrium, ovary, stomach, pancreas, small bowel, hepatobiliary tract, ureter, renal pelvis). |
|  | Muir-Torre syndrome | HNPCC + sebaceous adenomas and carcinomas, and keratocanthomas (skin lesions). |
|  | Turcot’s syndrome | HNPCC + glioblastomas (and multiple colonic adenomas). |
|  | Non-HNPCC and non-colonic tumors: endometrial,  ovarian, gastric, cervical, breast, skin, lung, prostate,  bladder tumors, glioma, leukemia, and lymphoma.  Fertility deficiency in mouse models. |  |
| Despite its role as an error-free repair  system, in certain circumstances, *MMR* proteins promote trinucleotide repeat expansion, a phenomenon associated with a number of neurological disorders in humans | Huntington’s disease | Brain disorder that causes movement (chorea, dystonia), cognitive (i.e. perseveration) and psychiatric disorders (obsessive-compulsive disorder, mania, bipolar disorder, social withdrawal, insomnia) |
|  | Myotonic dystrophy | Myotonia and progressive skeletal muscle weakness and wasting; cardiac conduction defects; insulin-resistance; testicular atrophy; respiratory insufficiency; cognitive impairment; premature cataract. |
|  | Fragile X syndrome  Fragile X associated Tremor/Ataxia Syndrome (FXTAS) and fragile X-associated primary ovarian insufficiency (FXPOI) | Mild to severe cognitive impairments in males but less severe cognitive impairment in females; elongated face; prominent ears; post-pubertal macroorchidism; cardiac anomalies (mitral valve prolapse); strabismus; seizures; poor eye contact; attention deficit hyperactivity disorder; flat feet; scoliosis etc. In full mutation males: severe obesity; hyperphagia; lack of satiation after meals; hypogonadism; delayed puberty. |
| ***HR repair [454-458]*** | | |
| **Defective gene** | **Related clinical syndrome** | **Specific clinical features** |
| *BRCA1, BRCA2* | Breast/ovarian cancer, adenocarcinoma of the colon |  |
| *NBS* | Nijmegen breakage syndrome | Short stature; microcephaly; distinctive facial features (sloping forehead, prominent nose, large ears, small jaw, upslanting palpebral fissures); recurrent respiratory tract infections; immunodeficiency (low IgG,IgA, T-cells); intellectual disability, increased (x 50) risk of cancer (non-Hodgkin lymphoma, medulloblastoma, glioma, rhabdomyosarcoma). |
| *BLM* | Bloom syndrome | Increased risk of cancer (any type that develop earlier in life) short stature; sun-sensitive skin changes on the face, hands and/or arms; a high-pitched voice; distinctive facial features including a long, narrow face, small lower jaw, large nose and prominent ears; learning disabilities; increased risk of diabetes; chronic obstructive pulmonary disease (COPD); recurrent infections of the upper respiratory tract, ears and lungs during infancy. |
| *Mre11/Rad50/NBS1* complex *(MRN)* | Ataxia telangiectasia-like disorder | Moderate cerebellar degeneration, mental deficiency; immunodeficiency (normal levels of total IgG, IgA and IgM, but reduced levels of specific functional antibodies); no telangiectasia; sensitivity to ionizing radiation; chromosomal instability; possibly, cancer predisposition (It is not known whether ATLD patients have a true predisposition to cancer as too few patients have been described so far); later onset of the neurological features, and slower progression (compared with AT). |
| *WRN* | Werner syndrome | Dramatic, rapid appearance of features associated with normal aging, after puberty: graying and loss of hair; a hoarse voice; thin, hardened skin; "bird-like" facial appearance; cataracts; skin ulcers; type 2 diabetes; diminished fertility; atherosclerosis; osteoporosis; multiple, rare types of cancer; thin arms and legs; thick trunk due to abnormal fat deposition. |
| *RAD51C* | Increased risk for breast and ovarian cancer |  |
| ***NHEJ [459-462]*** | | |
| **Defective gene** | **Related clinical syndrome** | **Specific clinical features** |
| *Ku70/80* | Autoantibodies against Ku have been found in scleroderma, SLE and other autoimmune disease |  |
| *DNA-PKc* | Significantly reduced levels of DNA-PKcs in the brain of patients with Alzheimer disease |  |
| *DNA ligase IV* | LIG4 syndrome | Radio-sensitivity; increased disposition to leukemia; cytopenias; growth retardation; developmental delay; microcephaly; facial dysmorphism. |
| *XRCC4* | Microcephalic primordial dwarfism | Microcephaly; facial dysmorphism; developmental delay; short stature. |
| *XLF* | XLF syndrome | Microcephaly; facial dysmorphism; growth retardation; severe combined immunodeficiency. |
| *Artemis* | Athabascan-type SCID | Severe combined immunodeficiency; individuals susceptible to radiation. |
| *Aprataxin* | Ataxia oculomotor apraxia-type 1 | Early onset of ataxia; oculomotor apraxia; chorea; myoclonus; peripheral neuropathy. |
| ***FANC mechanism [463-464]*** | | |
| **Defective gene** | **Related clinical syndrome** | **Specific clinical features** |
| Biallelic mutations in at least one of 13 different genes *FANCA, FANCB, FANCC, BRCA2 (FANCD1), FANCD2, FANCE, FANCF, FANCG, FANCI, BRIP1 (FANCJ), FANCL, FANCM* and *PALB2 (FANCN)* | Fanconi anaemia | ~50-fold increased risk of developing any cancer type, with striking susceptibility to acute myeloid leukemia and squamous cell carcinomas (SCC) of the head and neck (HNSCC) and gynecological tract; bone marrow failure; congenital defects; growth retardation; congenital malformations; learning disability; hyper-pigmentation; median life expectancy ≤20 years.  Note: biallelic carriers of BRCA2 (FANCD1) and PALB2 (FANCN) display a severe Fanconi anemia phenotype and solid tumor development. |
| Homozygous missense mutation in the *RAD51C* | FA-R syndrome (FA-like disorder) | Presence of characteristic congenital abnormalities and sensitivity of cells to ICL agents, without the early development of hematological abnormalities or cancer |
| Biallelic mutations in *BRCA1* | FA-S syndrome (FA-like disorder) | Early-onset of breast cancer; multiple developmental and cellular anomalies consistent with a new Fanconi anemia subtype. |
| Defects in the *FA/BRCA* pathway in the general, *non-FA* population |  |  |
| *FANCD1/BRCA2* and *RAD51C* mutations | Predisposition to breast and ovarian cancer |  |
| Carriers of *FANCJ* and *FANCN* mutations | Predisposition to breast cancer at lower penetrance |  |
| *FANCD1/BRCA2, FANCC* and other *FA* gene mutations | Predisposition to pancreatic cancer |  |
| ***ATM-mediated DDR signaling [465]*** | | |
| **Defective gene** | **Related clinical syndrome** | **Specific clinical features** |
| *ATM* | Ataxia-telangiectasia phenotype | Progressive cerebellar ataxia; gonadal atrophy; immune deficiencies; oculocutaneous telangiectasias; radiation sensitivity (abnormal cellular responses to ionizing irradiation, but normal responses to ultraviolet radiation); premature aging; increased risk of cancers, particularly lymphomas. |
| ***ATR-mediated DDR signaling [461]*** | | |
| **Defective gene** | **Related clinical syndrome** | **Specific clinical features** |
| *ATR* | Seckel syndrome | Intrauterine growth retardation; dwarfism; microcephaly; mental retardation; cells from these patients show enhanced sensitivity to UV radiation. |

**Abbreviations**

ATM: Ataxia-telangiectasia mutated;

ATR: Ataxia telangiectasia and Rad3-Related Protein;

BLM: Bloom syndrome RecQ like helicase;

BRCA1/2: Breast cancer 1/2; *BRIP1:* BRCA1 interacting protein C-terminal helicase 1;

CSA, CSB: Cockayne syndrome WD repeat protein A/B;

FA: Fanconi anemia;

FANC (A, B, C, D1/2, E, F, G, J, L, M): Fanconi anemia complementation group (A, B, C, D1/2, E, F, G, J, L, M);

HNPCC: Hereditary non-polyposis colorectal cancer;

hMLH1: MutL homolog 1;

hMSH2/6: MutS homolog 1;

Ku70/80: Lupus Ku autoantigen protein p70/80;

MCSZ disorder: Microcephaly, seizures, and developmental delay disorder;

MMR: Mismatch repair;

MUTYH: mutY DNA glycosylase;

NBS: Nijmegen breakage syndrome;

NTHL1: Nth Like DNA glycosylase 1;

OGG1: 8-Oxoguanine DNA glycosylase 1;

PALB2: Partner and localizer of BRCA2;

hPMS1/2: PMS1 homolog 1/2, mismatch repair system component;

PNKP: polynucleotide kinase 3'-phosphatase;

RAD51C: RAD51 paralog C;

TFIIH: Transcription factor II human;

WRN: Werner syndrome helicase;

XLF: synonymous for non-homologous end joining factor 1 (NHEJ1);

XPA, XPB, XPC, XPD, XPE, XPF, XPG: Xeroderma pigmentosum group (A,B,C,D,E,F,G)-complementing protein.
